# Mechanical Properties and Recyclability of Fiber Reinforced Polyester Composites

**DOI:** 10.1021/acssuschemeng.4c03341

**Published:** 2024-06-20

**Authors:** Eloise K. Billington, Theona Şucu, Michael P. Shaver

**Affiliations:** a Department of Materials, University of Manchester, Oxford Road, Manchester M13 9PL, U.K.; b Sustainable Materials Innovation Hub, Henry Royce Institute, University of Manchester, Manchester M13 9PL, U.K.

**Keywords:** Cyclic esters, Bioderived, Bis(1,3-dioxolan-4-one), Vitrimers, Vacuum-assisted resin infusion, Depolymerization

## Abstract

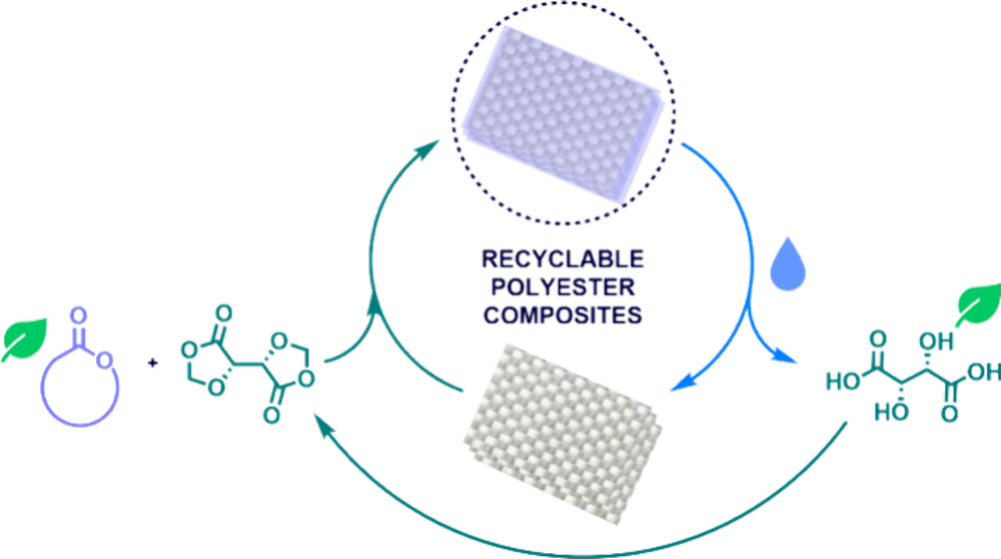

Fiber reinforced polymer composites (FRPs) are valuable construction materials owing to their strength, durability, and design flexibility; however, conventional FRPs utilize petroleum-based polymer matrices with limited recyclability. Furthermore, fiber reinforcements are made from nonrenewable feedstocks, through expensive and energy intensive processes, making recovery and reuse advantageous. Thus, FRPs that use biobased and degradable or reprocessable matrices would enable a more sustainable product, as both components can be recovered and reused. We previously developed a family of degradable and reprocessable cross-linked polyesters from bioderived cyclic esters (l-lactide, δ-valerolactone, and ε-caprolactone) copolymerized with a bis(1,3-dioxolan-4-one) cross-linker. We now incorporate these networks into FRPs and demonstrate degradability of the matrix into tartaric acid and oligomers, enabling recovery and reuse of the fiber reinforcement. Furthermore, the effect of varying comonomer structure, catalyst, reinforcement type, and lay-up method on mechanical properties of the resultant FRPs is explored. The FRPs produced have tensile strengths of up to 202 MPa and Young’s moduli up to 25 GPa, promising evidence that sustainable FRPs can rival the mechanical properties of conventional high performance FRPs.

## Abstract 2

This article details the synthesis and recycling of fiber-reinforced polyester composites, which are more sustainable than those currently used industrially.

## Introduction

Thermoplastics and thermosets are lightweight, durable, and nonconductive yet may lack sufficient strength, dimensional stability, or stiffness for load bearing applications.^1^ Additional strength can be imparted through reinforcement with fiber fillers to create fiber reinforced polymer composites (FRPs) which have a significantly higher strength to weight ratio.^2^ FRPs are incredibly versatile and can be used for a wide variety of applications, including aerospace and automotive components, boat hulls, sports equipment, and wind turbine blades.^3^

Despite their utility, there are major drawbacks associated with the use of FRPs, primarily the use of nonrenewable materials in their synthesis and the generation of vast quantities of nonrecyclable waste at end-of-life.^4^ FRPs are generally made using a cross-linked, thermosetting polymer matrix with elusive reprocessability, which precludes recycling and forces the majority of FRP waste to be landfilled, buried, or incinerated.^5,6^ A promising alternative to conventional thermosets as polymeric matrix materials, that offer greater scope for reprocessing, are covalent adaptable networks (CANs).^7,8^ These are thermosetting polymers that contain dynamic covalent bonds, such as ester, imine, disulfide, hindered urea, acetal, carbamate, or Diels–Alder linkages. These bonds undergo reversible exchange reactions when subjected to certain external stimuli, such as heat, solvent, or UV light.^9^ The mechanism of exchange can be associative or dissociative, with associative CANs often being referred to as vitrimers, a term coined by Leibler and collaborators.^10−15^

Our group has developed a bifunctional 1,3-dioxolan-4-one monomer, bis(1,3-dioxolan-4-one) (bisDOX),^16^ synthesized from l-(+)-tartaric acid; a nonhazardous, inexpensive, naturally occurring starting material.^17^ Through copolymerization of cyclic ester monomers l-lactide, δ-valerolactone, and ε-caprolactone with bisDOX as a cross-linker, poly(lactic acid), polyvalerolactone, and polycaprolactone networks (PLA, PVL, and PCL, respectively) have been made.^16^ These networks have high thermal stability and tunable mechanical properties, depending on the comonomer structure. Reprocessability is enabled by transesterification reactions at elevated temperatures, facilitated by the polymerization catalyst which remains embedded in the networks. Moreover, they are susceptible to degradation via base-catalyzed hydrolysis, facilitating recovery of oligomers, monomers, and l-(+)-tartaric acid. These thermosets showed promise as renewable and degradable alternatives to petroleum-derived thermosets.

**Figure 1:**
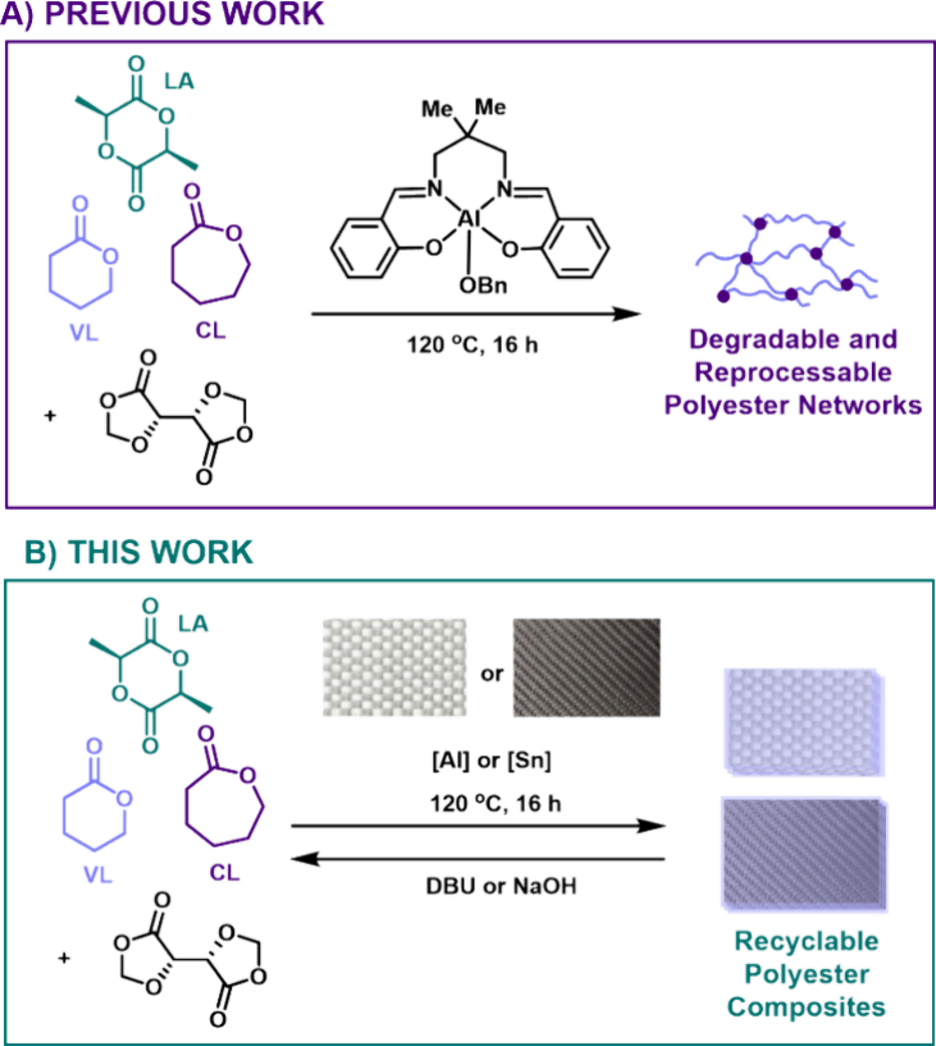
(A) Synthesis of degradable cross-linked polyester networks with δ-valerolactone (VL), l-lactide (LA), and ε-caprolactone (CL) monomers and bisDOX cross-linker and (B) development of fiber reinforced polymer composites.

This work focuses on the extension of these degradable polyester resins to support recyclable FRPs (Figure 1). We present a novel vacuum-assisted resin infusion process carried out under air-free conditions, explore the intersection between catalyst and monomer choice with layup procedure, and test the effects of fiber sizing, fiber reinforcement type (glass or carbon), and comonomer structure on composite performance. Finally, chemical recycling of the matrix and recovery and reuse of the fiber reinforcement are demonstrated.

## Materials and Methods

### General Considerations

All air-sensitive manipulations were performed under nitrogen in an MBraun LABmaster sp glovebox, using a dual-manifold Schlenk line equipped with an in-line gas drying column containing copper catalysts and molecular sieves or using a modified lay-up procedure (*vide infra*). Glassware used in air and moisture sensitive reactions was dried in an oven at 200 °C for a minimum of 12 h.

### Materials

The following chemicals were used as received: 2,2-dimethyl-1,3-propanediamine (99%, ACROS Organics), salicylaldehyde (99%, ACROS Organics), trimethylaluminum (Sigma-Aldrich), l-(+)-tartaric acid (Fisher Scientific), paraformaldehyde (97%, Alfa Aesar), *p*-toluenesulfonic acid monohydrate (Sigma-Aldrich), ethyltriethoxysilane (96%, Alfa Aesar), aminopropyltrihydroxysilane (ABCR GmbH), glycidoxypropyltriethoxysilane (97%, Fluorochem), hydroxymethyltriethoxysilane (50% in EtOH, Fluorochem), Joncryl ADR-4400 (BASF), and bisphenol A diglycidyl ether (Fluorochem). Anhydrous toluene was obtained from an MBraun 7 solvent purification system containing alumina and copper catalysts and degassed by three successive freeze–pump–thaw cycles prior to use. CDCl_3_ (99.8 atom % D, Sigma-Aldrich) was stirred over CaH_2_ overnight and distilled under an inert atmosphere before being stored over 3 Å molecular sieves. l-(+)-Lactide (98%, Corbion) was stored under vacuum at 25 °C overnight and then sublimed. Benzyl alcohol (99%, Fluorochem) and δ-valerolactone (99%, Fluorochem) were stirred over CaH_2_ (99.9%, Aldrich) and distilled under reduced pressure. Woven glass fabric (290 g cm^–3^ plain weave) and carbon fabric (290 g cm^–3^ 2 × 2 twill) were purchased from EasyComposites.

### Methods

#### Hand Lay-Up Methodology To Prepare Cross-Linked PVL/PCL Composites

In a nitrogen filled glovebox, δ-valerolactone (10 g, 99.9 mmol, 100 equiv), bisDOX (0.870 g, 4.99 mmol, 5 equiv), and [salen]AlOBn catalyst (0.442 g, 0.999 mmol, 1 equiv) were weighed into an oven-dried vial. The vial was then sealed and removed from the glovebox, and the contents were ultrasonicated for 5 min to ensure good mixing. They were then placed in a glovebag (Aldrich AtmosBag) and the contents degassed. The mixture was poured in a preheated Petri dish presprayed with a PTFE mold-release spray (Rocol), containing glass fiber mats. The samples were covered and left to cure for 16 h at 120 °C. The formed materials were then cooled and peeled off of the dishes.

#### VARI Methodology To Prepare Cross-Linked PVL/PCL Composites

In a nitrogen filled glovebox, δ-valerolactone (10 g, 99.9 mmol, 100 equiv), bisDOX (0.870 g, 4.99 mmol, 5 equiv), and [salen]AlOBn (0.442 g, 0.999 mmol, 1 equiv) or Sn(oct)_2_ catalyst (0.405 g, 0.999 mmol, 1 equiv) were weighed into an oven-dried vial. The vial was sealed and shaken vigorously to ensure good mixing. The mixture was transferred to a 25 mL SGE gastight syringe and removed from the glovebox. The syringe was connected to inlet tubing of a VARI setup, containing glass fiber mats, and the mixture was injected. The sample was left to cure for 16 h at 120 °C in a Binder FD115 convection oven, then cooled and removed from the VARI setup.

#### Hot Press Methodology To Prepare Cross-Linked PLA Composites

In a nitrogen filled glovebox, l-lactide (10 g, 69.4 mmol, 100 equiv), bisDOX (0.603 g, 3.47 mmol, 5 equiv), and [salen]AlOBn (0.306 g, 0.693 mmol, 1 equiv) or Sn(oct)_2_ catalyst (0.281 g, 0.693 mmol, 1 equiv) were weighed into an oven-dried vial and mixed thoroughly, and then the mixture was spread over glass fiber mats. The mats were placed between two pieces of Nylon film, sealed together with vacuum sealant tape, and removed from the glovebox. The sample was hot pressed at 120 °C for 2 h and then left to cure for 16 h at 120 °C before being cooled and removed from between the nylon sheets.

#### Representative Procedure for Glass Fiber Modification

Glass fiber mats were annealed in a furnace at 500 °C for 2 h. A solution of organofunctional triethoxysilane was diluted with H_2_O to make a 50 wt % solution. Acetic acid was added until pH 4–5 was reached, and then the solution was stirred for 30 min at room temperature. The solution was diluted with H_2_O to make a 10 wt % solution of the hydrolyzed silane, and the annealed glass fiber mats were soaked in the solution for 2 h at room temperature. After this, the solution was decanted, and the glass fibers were dried in a vacuum oven at 40 °C for 48 h.

#### Representative Procedure for Woven Fiber Mat Recycling

Rectangular composite samples were immersed in a 1 M solution of DBU in acetonitrile (MeCN) and left to soak for 1 h, in which time the matrix was degraded. The solutions were then decanted, and the fiber mats were washed with deionized water and soaked for 30 min. 1 M HCl was added to the solution until pH = 7 was reached, and the fiber mats were rinsed until the washings were a constant pH. Finally, the mats were washed with acetone and left to dry in a vacuum oven at 40 °C overnight, prior to reuse.

All other procedures are included in the Supporting Information.

### Instrumentation

NMR experiments were performed at 298 K on either a 400 MHz Bruker AVIII spectrometer or a 500 MHz Bruker AVIII HD spectrometer. Chemical shifts are reported as δ in parts per million (ppm) and referenced to the chemical shift of the residual solvent resonances (CDCl_3_^1^H δ = 7.26 ppm, ^13^C δ = 77.16 ppm) The resonance multiplicities are described as s (singlet), d (doublet), t (triplet), q (quartet), or m (multiplet). Thermogravimetric analysis (TGA) was performed using a TA Instruments Q800 instrument. The samples (10–25 mg) were heated under nitrogen gas from room temperature to 600 °C at a rate of 10 °C min^–1^. All analyses were performed in triplicate. Filler contents were calculated using eqs 1 and 2, along with the value for the char content of **PVL-Al** at 600 °C (2.7%).
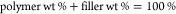
1
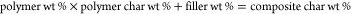
2

Differential scanning calorimetry (DSC) was performed on a DSC 2500 TA Instruments using heat (−80 to 200 °C)/cool (from −80 to −80 °C)/heat (from −80 to 200 °C) cycles at a rate of 10 °C min^–1^. Values of *T*_g_ and *T*_m_ were obtained from a second heating scan. All analyses were performed in triplicate. Tensile tests were conducted on a static testing Instron 3344L3928 fitted with a 2 kN load cell. The first-generation samples, matching ISO 527-2-1BB, and second generation, matching ISO 527-4-3, were tested at constant speeds of 200 mm/min. The strain at break (ε_b_), stress at break (σ_b_), and Young’s modulus (*E*) were measured and calculated. A minimum of 5 samples were tested per sample batch in accordance with the ISO standard used. The first-generation samples were obtained using an ISO-37-3 die cutter mounted on an 8 kN toggle press prior to analysis, ensuring the warp was parallel to the cutting direction. The second-generation samples were obtained by using a CNC diamond cutter. SEM imaging was performed using an FEI Quanta 250 FEG-SEM with an Oxford Instrument EDS and GATAN 3view system. Prior to analysis, the composite samples were mounted on aluminum stubs with a carbon tab, using conductive carbon tape. The samples were then coated using an 80/20 Au/Pd alloy using a Quorum Au/Pd coater to ensure satisfactory conductivity.

## Results and Discussion

The bifunctional cross-linker, bisDOX, can be copolymerized with cyclic ester monomers to afford cross-linked polymeric networks, using the salen aluminum alkoxide catalyst, [salen]AlOBn.^16^ This work extended to using these resins in all-polyester FRPs (Figure 1). Composite nomenclature throughout the paper will reference the polyester being cross-linked (PLA, PCL, or PVL) followed by an indicator of the transesterification catalyst used in network formation (e.g., PVL-Al for [salen]AlOBn catalyzed synthesis), followed by the suffix -GF or -CF to indicate the nature of the reinforcement (e.g., PVL-Al-GF refers to a glass-fiber reinforced PVL composite prepared with an aluminum catalyst). Importantly, the Al-catalyzed reactions are air-sensitive, requiring methodological modifications from classical lay-up processes.

### Hand Lay-Up

**Figure 2:**
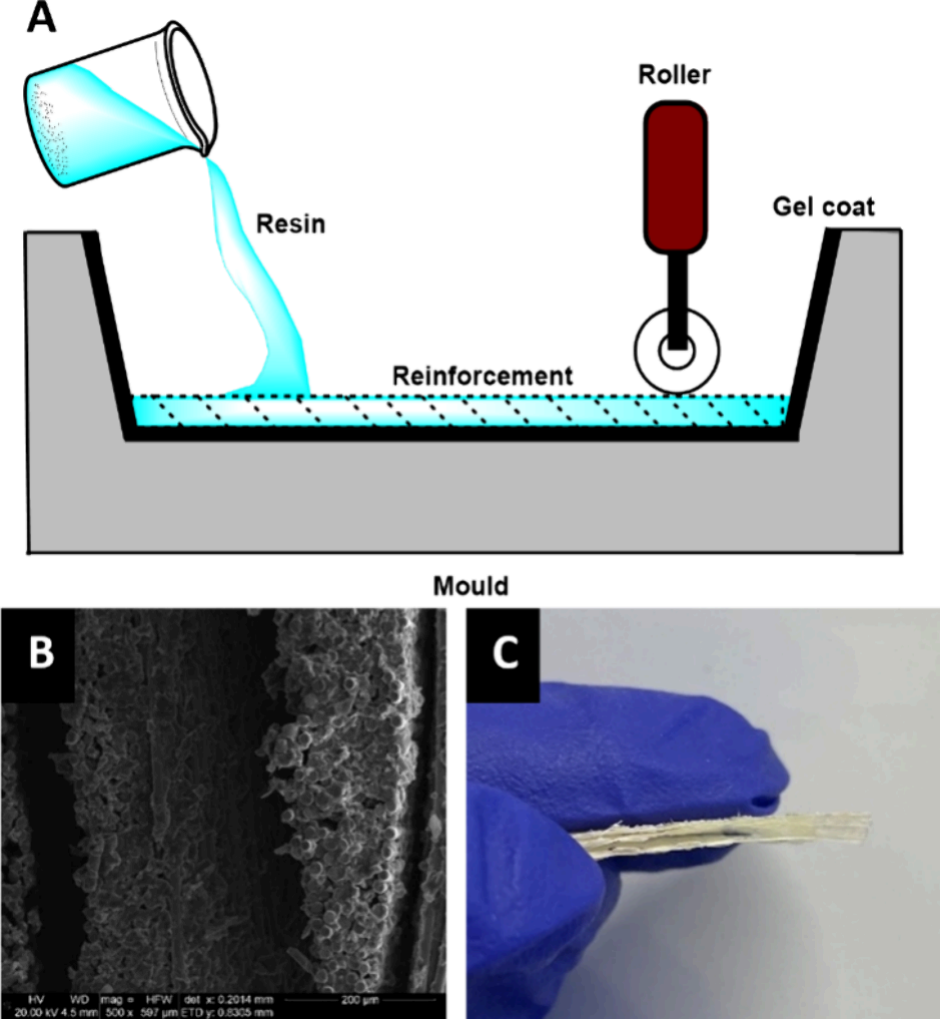
(A) Hand lay-up method of composite production, (B) SEM micrograph, and (C) photograph of PVL-Al-3GF composite made by hand lay-up.

Our first generation of GFRPs was prepared using hand lay-up, the oldest and still most common lay-up technique.^2^ It entails manually layering fibers into a mold, adding the matrix material, and then using a brush or roller to ensure uniform distribution of the resin and remove trapped air (Figure 2, top). Finally, the composite is left to cure at a specific temperature, before it is removed from the mold.^18^ This technique was modified to make it compatible with our air-sensitive catalyst by carrying it out under an inert atmosphere. However, there were challenges with this approach, which affected the integrity of the composites produced. Irregular resin distribution within and between composite samples stemmed from difficulties maintaining consistent resin thickness when pouring it over the fibers in a glovebag while poor fiber wetting left the composites with a distinct layer of resin on top of the fiber mats, leaving large voids between layers which resulted in delamination (Figure 2B,C).

### Inert VARI

**Figure 3:**
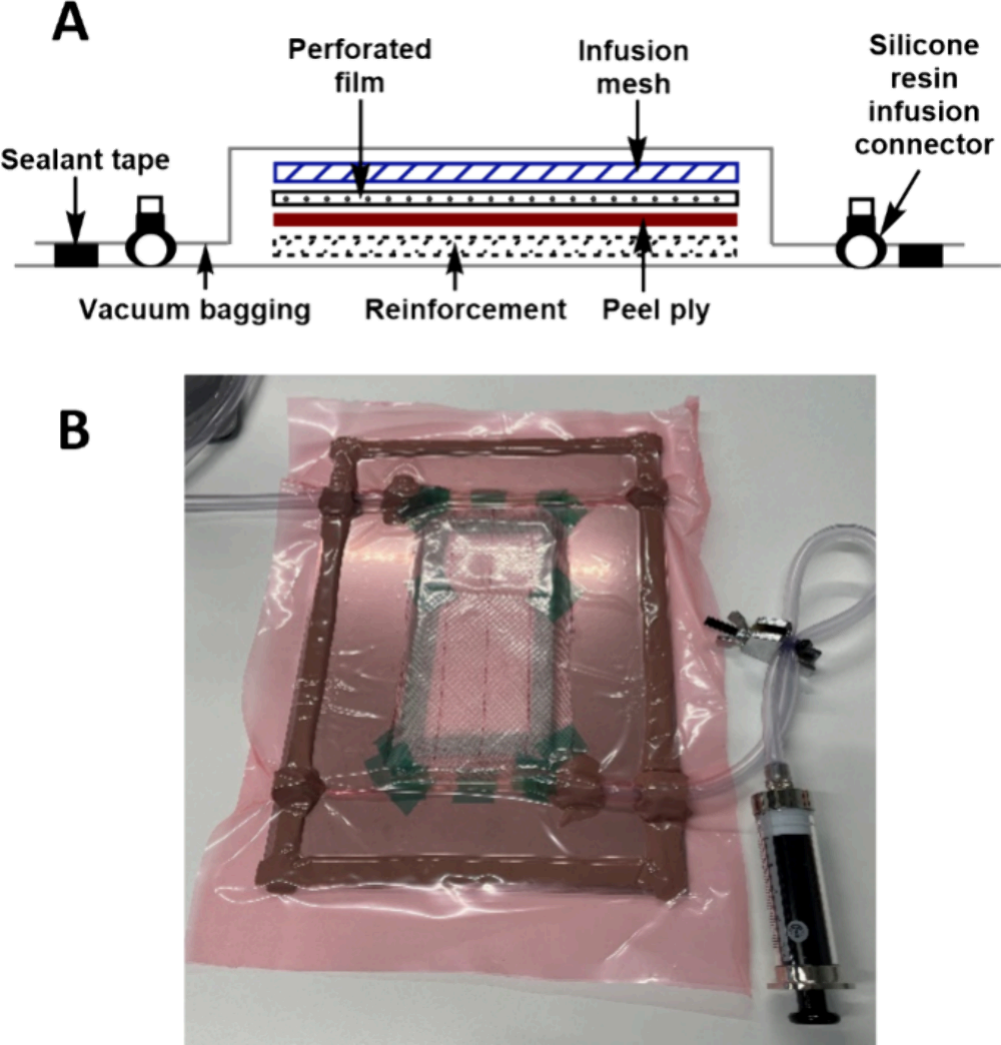
(A) Vacuum-assisted resin infusion (VARI) method of composite production and (B) modified VARI setup.

To improve fiber wetting in our composites, we turned to vacuum assisted resin infusion (VARI) where driving the liquid resin through a dry reinforcement under vacuum can improve performance (Figure 3; see the Supporting Information for more details).^19^ Typically, VARI is carried out on the benchtop; however, our air-sensitive catalyst had to be handled under an inert atmosphere, which presented practical difficulties. Recreating the VARI setup inside a glovebox or glovebag was challenging. Controlling the rate of resin flow through the VARI setup was difficult as manual dexterity was significantly reduced and efforts to increase the viscosity of the resin mixture by preinitiating the polymerization gave negligible improvements. Hence, when developing the VARI technique, we recognized the need to manufacture the composites outside of a glovebox without exposing the resin mixture to air. This was achieved through removing of the mixture from the glovebox in a gastight syringe and directly injecting it into the VARI setup (Figure 3, bottom). This modification proved highly successful, facilitating greater control over resin flow through the setup, improving fiber wetting and, consequently, performance (Figure S1).

### Optimization of Polyester Composites

Initial system optimization was conducted with the δ-VL monomer. Variables for optimization are discussed in this section, with characterization across these optimizations discussed in the characterization section.

#### GF Modification

**Figure 4:**
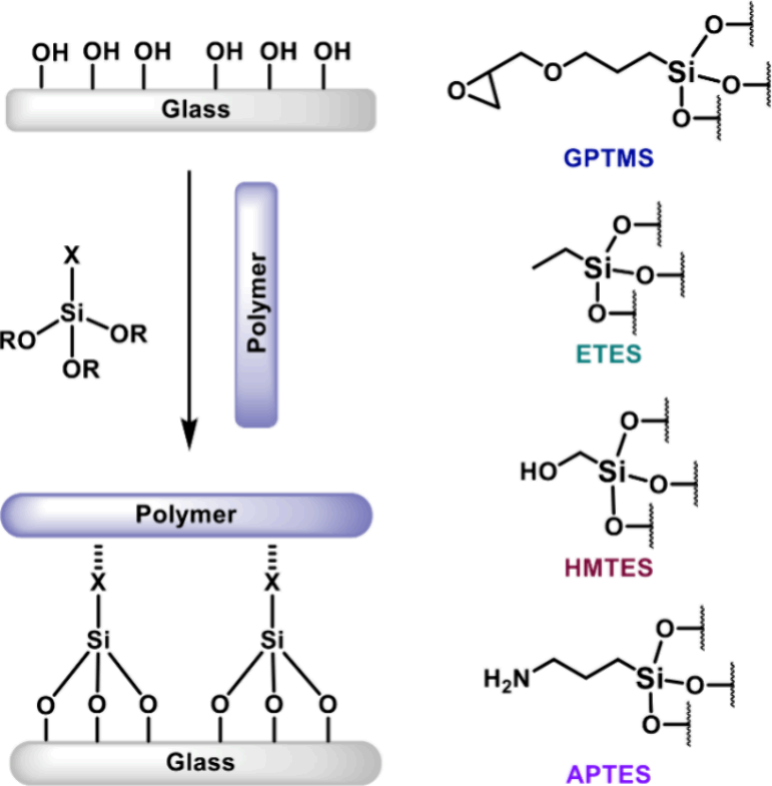
Schematic depicting functionalization of glass fiber surface with an organosilane (left). Structure of glass fibers functionalized with γ-glycidoxypropyltrimethoxysilane (GPTMS), ethyltriethoxysilane (ETES), γ- hydroxymethyltriethoxysilane (HMTES), and aminopropyltriethoxysilane (APTES) (right).

To improve the interfacial adhesion between the GFs and matrix, a surface treatment, or “size”, is often applied to GFs. This size is bifunctional; one part of its structure interacts with the matrix and the other with the GF, so it acts as a coupling agent. The formation of stronger noncovalent, or preferably covalent, connectivity between the GFs and matrix enhances the strength of interfacial bonding. This improves the efficiency of stress transfer between the matrix and GFs, thereby improving the mechanical properties of the FRP.^20^ The most common GF sizes are organosilanes bearing silanol groups, which condense with hydroxyl groups on the surface of the GFs, and variable groups that interact with the polymer matrix (Figure 4).^21,22^ We investigated sizing with four silanes: γ-glycidoxypropyltrimethoxysilane (GPTMS), ethyltriethoxysilane (ETES), γ-hydroxymethyltriethoxysilane (HMTES), and aminopropyltriethoxysilane (APTES). While GF modification did not improve the mechanical properties of the composites in these PVL systems, this optimization will prove important in other systems (*vide infra*). For PVL, Young’s modulus was unaffected, while strain at break (ε_b_) and stress at break (σ_b_) decreased (Figure S2); the modification process involved soaking the fiber mats, which may have affected the integrity of the weave.

#### Matrix Modification

For construction applications, epoxy composites are often preferred to polyester composites, due to their superior thermomechanical properties.^23^ We hoped to increase the strength and toughness of our polyester systems and make them more competitive with epoxies, by exploiting our understanding of the chemical dynamics of transesterification. These dynamics are exemplified during the mechanical recycling of PET.^24,25^ The low *T*_g_ of the polyester networks, combined with the low *E*_a_ for transesterification, enables vitrimeric exchanges to occur at ambient temperature, which could reduce the structural integrity of the resins. We postulated that by using epoxy-based chain extension chemistry, we could balance the dynamic network structure with that of a conventional thermoset. Thus, the commercial chain extender Joncryl ADR-4400 was added to the matrix mixture to introduce a small number of nondynamic cross-links (Scheme S1) in addition to the dynamic cross-links. However, when GF was added to make a composite (**PVL-Al-J-GF**), the resin did not set. The hydroxyl groups on the GF surface can open the epoxy ring in Joncryl, preventing reaction with the secondary hydroxyl groups in the ring-opened bisDOX and inhibiting the polyester network formation.

To circumvent this issue, GFs were functionalized with ETES (EtmGF) to mask the reactive hydroxy groups. Composites with EtmGF and Joncryl (**PVL-Al-J-EtmGF**) were prepared, resulting in materials with had the highest Young’s modulus (1.43 GPa) of all of the GF-reinforced PVL composites made using hand lay-up (Table S1), demonstrating the utility of Joncryl for improving the mechanical properties of this system.

#### Variation of Fiber Reinforcement

Carbon fibers and their composites exhibit superior mechanical properties than their glass fiber counterparts, so they yield more robust construction materials. Initially, **PVL-Al-3CF** was made using the VARI methodology. It was observed that CF mats absorbed more resin into their weave structure than GF mats, so a larger quantity of resin was needed to adhere the mats together and avoid delamination, corroborated via SEM analysis (cf. Characterization).

#### Variation of Comonomer

PCL is made from ε-caprolactone, which, like δ-valerolactone, is a liquid monomer, so the same VARI procedure was used for the setup of both PCL and PVL composites. In contrast, PLA is made using a solid monomer, l-(+)-lactide, which precluded the use of VARI, as this technique is only suitable for liquid resin mixtures under ambient conditions. Thus, alternative methods have to be investigated. The most successful approach was a compression molding procedure, which entailed mixing the resin components in the glovebox and spreading the mixture onto GF mats, which were then sandwiched between two sheets of vacuum bagging. Once this setup was sealed, it was transferred outside the glovebox to then be hot pressed, before leaving to cure in the oven.

#### Alternative Catalysis

While the Al-salen system is a proven catalyst for the ring-opening polymerization (ROP) of DOX monomers,^26^ the air-sensitive nature would make scale-up challenging. Sn(oct)_2_ is an effective catalyst for the ring-opening polymerization (ROP) of our cyclic ester comonomers,^27−29^ although it does not work as effectively on DOX systems. The resultant cross-linked networks (**PVL-Sn**, **PLA-Sn**, and **PCL-Sn**) showed lower gel contents than their salen-catalyzed counterparts (84 and 75% for **PCL-Sn** and **PVL-Sn** cf. 92 and 79% for **PCL-Al** and **PVL-Al**, respectively), indicative of a lower cross-link density. This was reflected in the mechanical properties of the GF composites (Table 1). In contrast, CF composites demonstrated better mechanical properties when Sn(oct)_2_ was used as the ROP catalyst (cf. Characterization), which will enable more facile scale up.

**Table 1:** Mechanical Properties of **PVL-Al**, **PCL-Al**, **PLA-Al**, **PVL-Sn**, and **PLA-Sn** Composites with Glass and Carbon Fiber Reinforcements, Manufactured via VARI^a^

sample	σ_b_ (MPa)^a^	ε_b_ (%)^a^	*E* (GPa)^a^	fiber content (%)^b^
**PVL-Al-3GF**	81.9 ± 7.6	1.51 ± 0.14	14.4 ± 1.9	77.0 ± 0.4
**PCL-Al-3GF**	70.6 ± 0.4	0.809 ± 0.093	15.3 ± 0.9	70.7 ± 1.7
**PLA-Al-3GF**	112 ± 35	3.21 ± 0.19	13.7 ± 1.4	64.8 ± 1.3
**PVL-Sn-3GF**	41.1 ± 2.8	1.03 ± 0.05	7.42 ± 0.25	73.7 ± 0.4
**PLA-Sn-3GF**	196 ± 27	2.49 ± 0.32	16.0 ± 1.8	71.3 ± 2.0
**PVL-Al-3CF**	41.3 ± 10.0	0.474 ± 0.045	15.9 ± 2.1	65.3 ± 0.5
**PLA-Al-3CF**	202 ± 15	1.75 ± 0.21	24.5 ± 1.7	52.5 ± 1.3
**PVL-Sn-3CF**	41.1 ± 1.7	0.603 ± 0.039	19.9 ± 2.8	56.3 ± 0.9
**PLA-Sn-3CF**	63.2 ± 14.2	2.48 ± 1.20	17.7 ± 2.5	59.2 ± 2.4

a σ_b_ (stress at break), ε_b_ (strain at break), and *E* (Young’s modulus) data obtained from tensile testing measurements.

b Fiber content obtained from TGA analyses.

### Characterization

The thermal properties of the composites were examined via TGA and DSC (Table S2, Figures S4–S15). TGA data showed that the GFRPs had fiber contents from 64.8% to 77.0%. This variation was attributed to differences in the amount of resin mixture remaining in the VARI setup. CFRPs had lower fiber contents than GFRPs, with values between 52.5% and 65.3%. This difference was due to the larger quantity of matrix mixture required to adhere the CF mats together (cf. Variation of Fiber Reinforcement).

Although the TGA data were comparable for each of the resins used, there were significant differences in the DSC thermograms. The **PLA-Al** composites showed a melting transition (*T*_m_) on the first heating cycle and a glass transition (*T*_g_) on all cycles, but no other thermal events were observed. The cross-linked structure in **PLA-Al** inhibits reformation of crystalline subdomains (on a DSC time scale) during repeated heat–cool cycles; thus, it no longer shows melting and crystallization transitions expected in linear polymers. The **PVL-Al** and **PCL-Al** composites, meanwhile, showed both melting and crystallization events, although their magnitudes and temperatures were decreased compared with comparative homopolymers; the lower crystallinity of the cross-linked matrix in these two systems may increase flexibility of linear chain segments and induce fewer interchain interactions, allowing for observations of *T*_m_ in each cycle.

**Figure 5:**
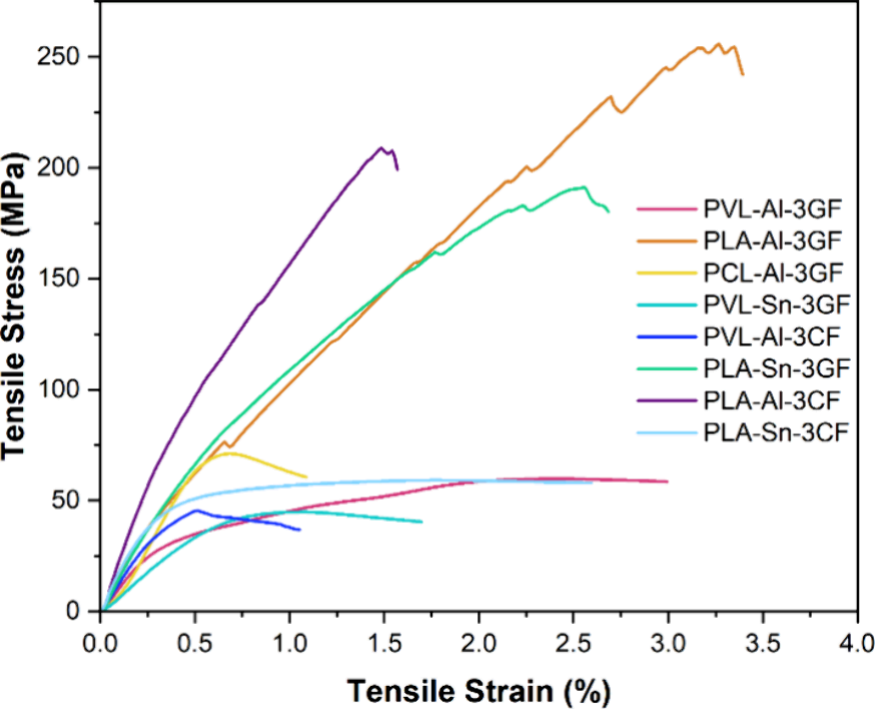
Tensile plots for PVL, PCL, and PLA FRPs, manufactured via VARI.

Tensile testing was used to measure the mechanical properties of the FRPs (Table 1, Figure 5). **PVL-Al** had demonstrated slightly better mechanical properties than **PCL-Al**, so it was expected that this behavior was translated to their corresponding composites. Indeed, ε_b_ and σ_b_ values were lower for **PCL-Al-3GF** (0.81% and 70.6 MPa) than for **PVL-Al-3GF** (1.51% and 81.9 MPa). Mirroring the properties of the homopolymers, **PLA-Al-3GF** had significantly higher σ_b_ and ε_b_ values (3.21% and 112 MPa). Across all three systems, Young’s moduli were not significantly different (14.4 GPa for **PVL-Al-3GF** vs 15.3 GPa for **PCL-Al-3GF** vs 13.7 GPa for **PLA-Al-3GF**). As the GF is the main component of the composites, the fibers dominated the behavior of the materials in the region of elastic deformation, minimizing matrix effects until the plastic region.

When GF were exchanged for CF, it was expected that the mechanical properties of the FRPs would increase, as CF have a higher tensile strength than GF.^2^ However, this was not always the case; the ε_b_ and σ_b_ of **PVL-Al-3CF** (0.474% and 41.3 MPa) were lower than its GF counterpart and its Young’s modulus (15.9 GPa) was not significantly different. This was attributed to the mode of failure of these composites, which underwent delamination instead of fiber breakage (Figure S3). As the composites delaminated before they were subjected to the amount of force required to break the fibers, the mechanical properties were reflective of the interfacial adhesion strength between the fibers and matrix, not of the reinforcement type used. In contrast, **PLA-Al-3CF** had a higher σ_b_ and Young’s modulus than its GF counterpart (202 MPa and 24.5 GPa, respectively), because the mode of failure was breakage. Substitution of the catalyst from [salen]AlOBn to Sn(oct)_2_ had a significant impact on the mechanical properties of the composites produced, as resins synthesized using Sn had lower cross-link densities. For this reason, **PVL-Sn-3GF** had lower Young’s modus, σ_b_ and ε_b_ (7.42 GPa, 41.1 MPa and 1.03%) than **PVL-Al-3GF**. Unexpectedly, **PVL-Sn-3CF** had a higher Young’s modulus than its salen counterpart (19.9 GPa vs 15.9 GPa), as the CF surface is inert while the GF surface is decorated with reactive −OH groups that may inhibit Sn-catalyzed transesterification. Overall, our highest performing composites (those with **PLA** matrices) achieved superior mechanical properties compared with other polyester systems reported in the literature,^30−33^ which makes them a promising sustainable alternative to those currently used in industry.

**Figure 6:**
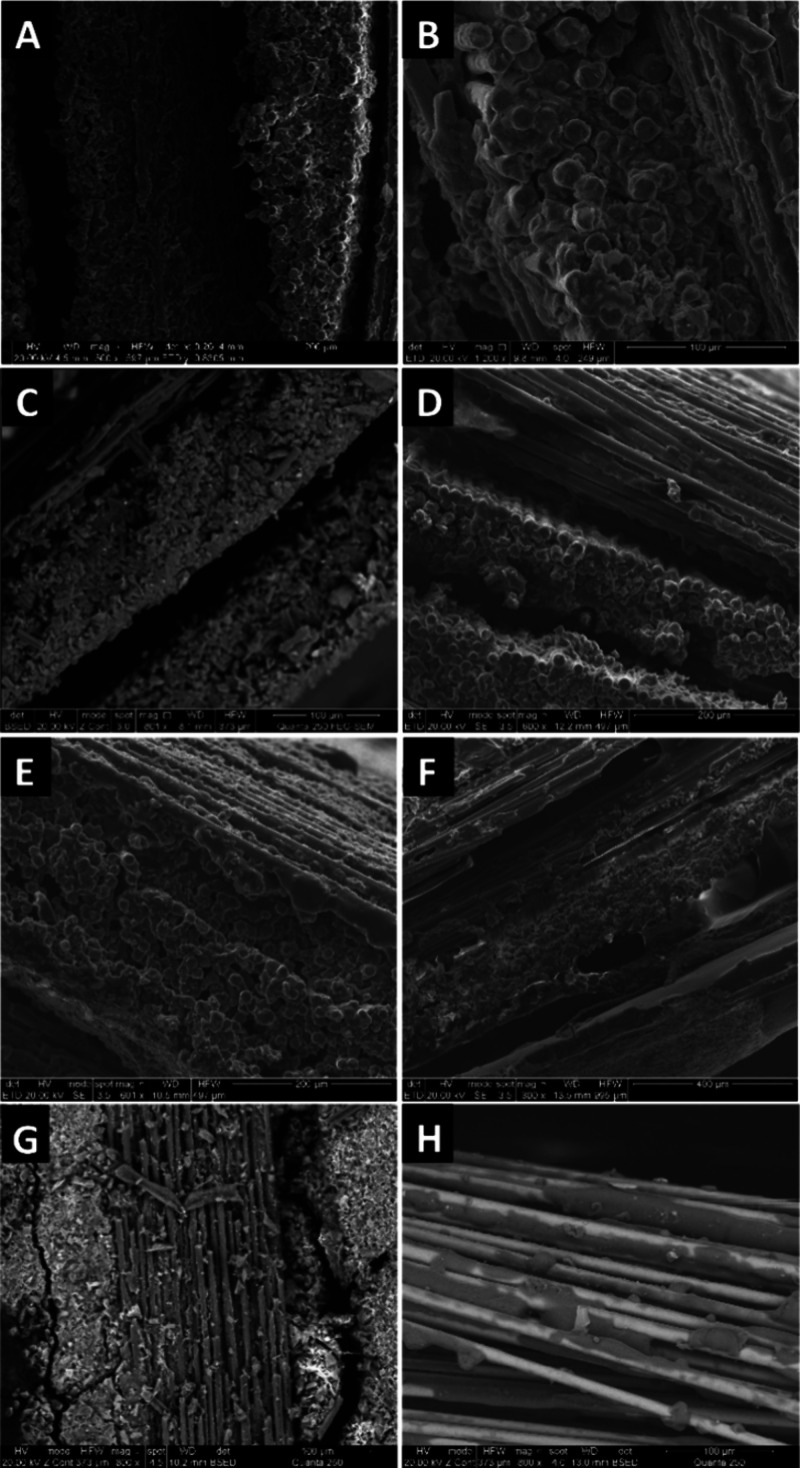
SEM images of (A) **PVL-Al-3GF** manufactured via hand lay-up, (B) **PVL-Al-3GF** manufactured via vacuum-assisted resin infusion, (C) **PVL-Al-3CF**, (D) **PVL-Sn-3GF**, (E) **PCL-Al-3GF**, (F) **PLA-Al-3GF**, (G) **PLA-Sn-3GF**, and (H) **PLA-Al**-**3CF.**.

Scanning electron microscopy (SEM) was used to gain deeper insight into the macroscopic morphology of the FRPs (Figure 6). FRPs manufactured using VARI had smaller interlaminar voids compared to hand lay-up methods, which accounts for their reduced delamination. When GFRPs were compared with CFRPs (e.g., **PVL-Al-3GF** and **PVL-Al-3CF**), it was evident that the latter had larger interlaminar voids, while intralaminar fiber wetting was improved. This was consistent with the experimental finding that when CF mats were used, larger quantities of resin were required, as the mats were more absorbent. There was no visible difference in macrostructure when the ROP catalyst was changed from [salen]AlOBn to Sn(oct_2_) (e.g., **PVL-Al-3GF** and **PVL-Sn-3GF**), whereas variation of comonomer structure had a noticeable impact. While **PCL-Al-3GF** had a very similar macrostructure to **PVL-Al-3GF**, **PLA-Al-3GF** showed significant differences, which was expected given the differences in the physical state of the monomers and the setup procedures (liquid VARI vs solid hot-pressing) used. **PLA-Al-3GF** had better fiber wetting, both in terms of the mats being well wetted and individual fibers being well coated. This, along with their superior mechanical properties, suggested that PLA composites had the strongest interfacial adhesion. This could be because PLA is more hydrophilic than PVL or PCL making it more compatible with the hydrophilic GF surface.

### Composite Recycling

**Figure 7:**
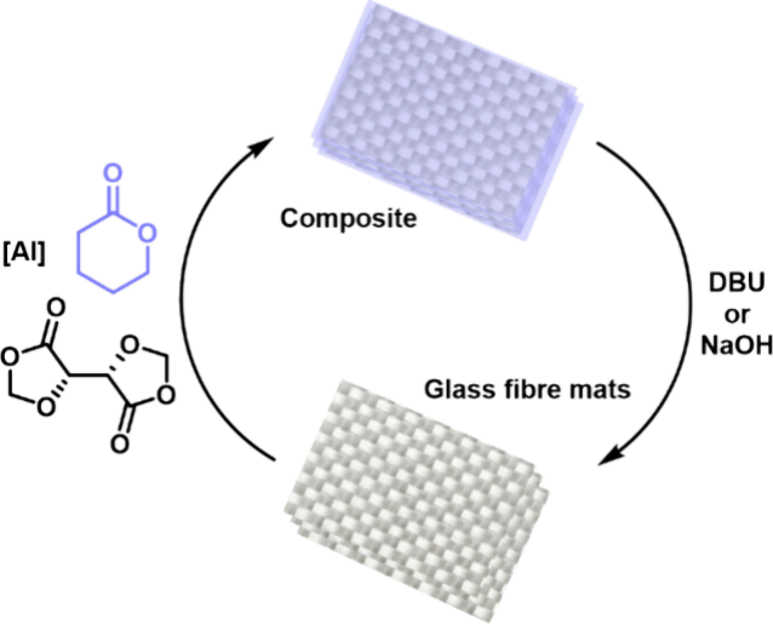
Recycling of **PVL-Al-GF** composites.

Finally, we turned our attention to demonstrating the recyclability of these composites (Figure 7). As previously established, these cross-linked polyesters are both degradable and reprocessable.^16^ Introduction of a second component (the reinforcement fibers) added another layer of complexity, both in terms of the technical challenge and economic drivers. In particular, CFs are very expensive and energy intensive to produce,^34^ so the ability to recover and reuse them is highly desirable. The cross-linked polyester networks described in this work can either be degraded in 12 h using aqueous NaOH or accelerated using an organic base such as 1,8-diazabicyclo[5.4.0]undec-7-ene (DBU), which reduces the time for complete depolymerization to just 30 min. This is significantly faster than for other systems presented in the literature, which can take days to weeks to depolymerise.^30^ Furthermore, unlike other processes,^35,36^ no heating is involved, which vastly reduces the energy requirements. When used to degrade the matrix of our polyester composites, these conditions allow recovery of the fiber reinforcement, along with oligomeric polyesters and tartaric acid (Figure S16) as three feedstocks for genuinely circular composites.

While depolymerization of the parent resins can be accelerated with agitation, this creates a challenge for recycling FRPs. The woven structure of the fiber mats is easily lost, which impacts the mechanical properties of composites made using recycled fibers, especially prevalent in CF mats.^37^ In order to facilitate retention of fiber architecture, recycling methods must minimize disturbance to the mats, so soaking treatments are preferable. The rapid DBU depolymerization is thus proposed as a more applicable method for future deployment and scale.

**Figure 8:**
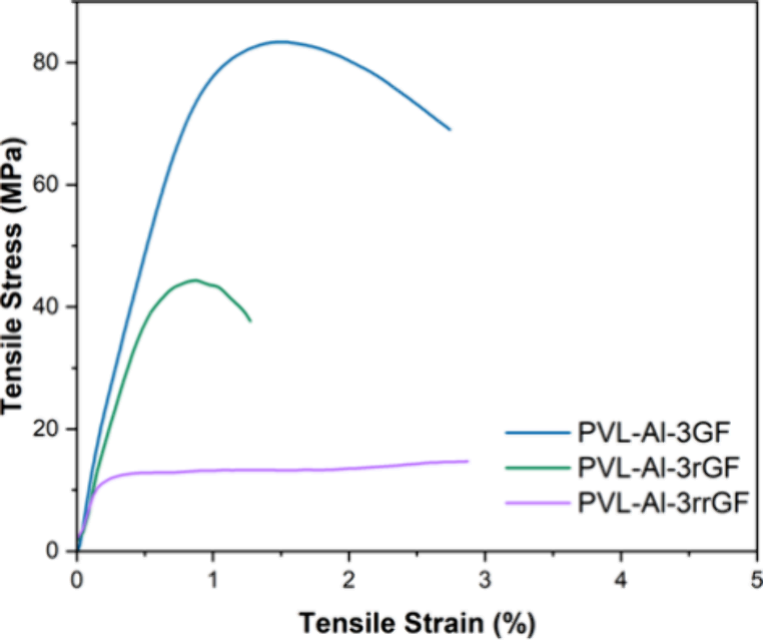
Tensile plot for GFRPs with once and twice recycled GFs, manufactured via VARI.

In the case of GF mats, depolymerization had minimal effect on the integrity of the weave and the resultant properties. However, with CF mats, the weave opened noticeably, despite having the same density as the GF mats. While it was expected that this could cause a drop in the mechanical properties of the resultant CF composite, the stress at the break actually increased. We hypothesize that as the original composite had bidirectional fibers (at 90°), the disturbance in the weave in the recycled composite resulted in fibers being more randomly aligned. In addition, increased permeability of the resin mixture into the mat improved fiber wetting, both of which can increase tensile strength. In contrast, one recycling cycle did not significantly change fiber alignment within GF mats, so the mechanical properties of **PVL-Al-3rGF** were relatively constant (Figure 8). Overall, it can be concluded that these FRPs can be recycled with minimal effects on their mechanical properties, as they were on the same order of magnitude as the originals.

**Figure 9:**
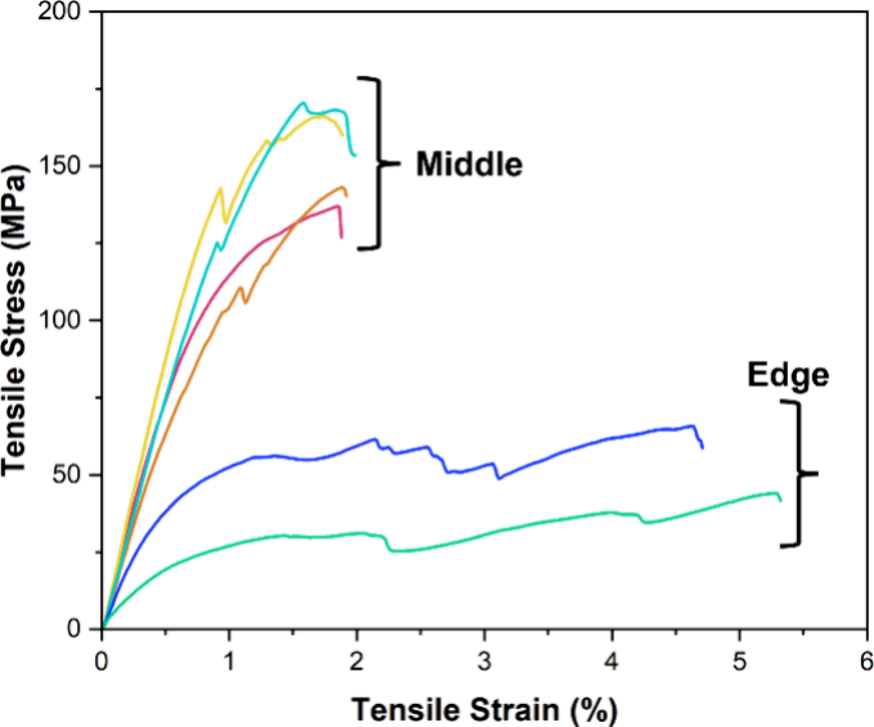
Tensile plot for **PLA-Al-3rCF**, manufactured via compression molding, indicating samples cut from the middle and the edge of the rectangular sheet.

We postulated that the effect of recycling on the weave of fiber mats would be reduced by recycling larger samples, as deterioration was more significant at the edges, and by using a larger composite, a smaller proportion of the total area would be at the edge. Thus, we synthesized a **PLA-Al-3rCF** composite that was 6 times wider than those previously used in our recycling studies (12 cm vs 2 cm). Tensile testing corroborated our theory, as there was a clear decrease in the mechanical properties of the edge samples in comparison with the middle samples (Figure 9, Figure S16). Furthermore, properties of the middle samples were more comparable with those of the original composite (Table S3, Figure S17).

**Figure 10:**
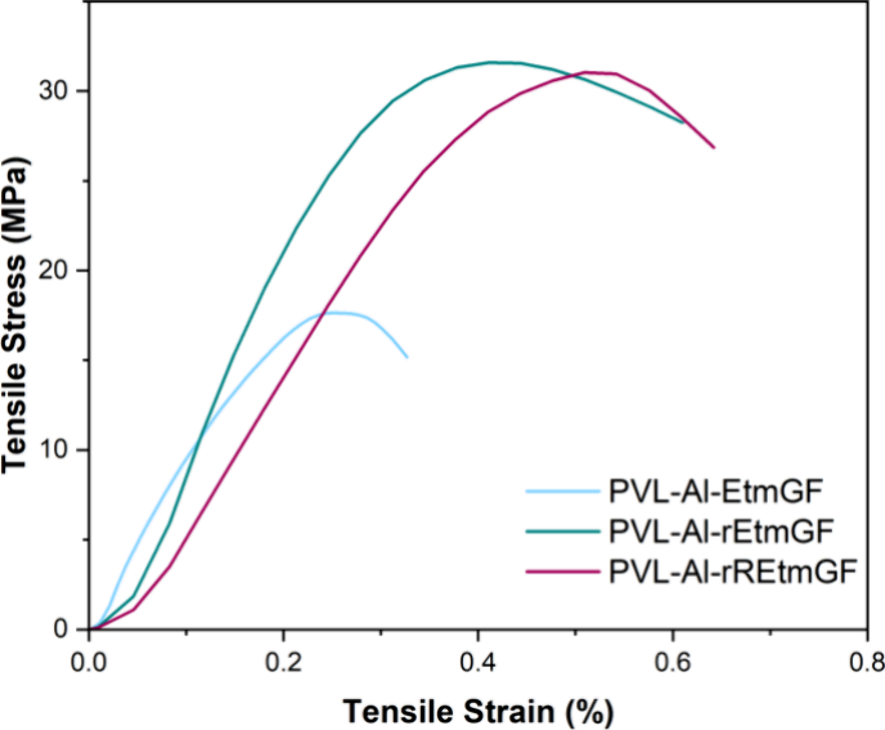
Tensile plot for ETES treated GFRPs, manufactured via VARI.

Another consideration when recycling FRPs is whether GF sizing has to be reapplied, or if it is unaffected by the conditions used to depolymerize the matrix. To investigate this, a sample of **PVL-Al-EtmGF** was split. Both halves were treated with DBU to depolymerize the matrix, then one set of GF (rEtmGF) was used directly to make **PVL-Al-rEtmGF**, and the other was retreated with ETES (to give rrEtmGF) prior to being made into **PVL-Al-rREtmGF**. The mechanical properties (Figure 10) of the resultant composites were not significantly different, so it was concluded that retreatment was not required. This was confirmed by TGA, as the rEtmGF showed 0.8% mass loss when heated to 800 °C, which was attributed to the loss of silane surface functionality.

## Conclusions

Cross-linked polyester composites from δ-valerolactone, l-lactide, and ε-caprolactone monomers and a bis(1,3-dioxolan-4-one) cross-linker with glass and carbon fiber reinforcements are promising sustainable composites. Two setup procedures suitable for use with air-sensitive resins were developed, modifying VARI and hot press technologies. Optimization of composite composition allowed tuning of the mechanical properties. Variation of the monomer structure, catalyst, and reinforcement changed strength and rigidity, with fully biobased PLA composites proving to be the strongest. The ability to improve the compatibility between matrix and reinforcement via functionalization of the glass fiber surface and the addition of classical recycling additives was established. Finally, the recycling of these composites was demonstrated through degradation of the matrix under accelerated basic hydrolysis conditions, followed by recovery and reuse of the fiber reinforcement. These materials present an encouraging step in the transition toward more sustainable FRPs.

## Supporting Information Available

Materials, methods, additional data, and further experimental details (PDF)

## Abbreviations

bisDOX: bis(1,3-dioxolan-4-one)
CAN: covalent adaptable network
CFRP: carbon fiber reinforced composite
DSC: differential scanning calorimetry
FRP: fiber reinforced polymer
GFRP: glass fiber reinforced polymer
PCL: polycaprolactone
PLA: poly(lactic acid)
PVL: polyvalerolactone
ROP: ring opening polymerization
SEM: scanning electron microscopy
TGA: thermogravimetric analysis
