# Supplementary material for: Mechanical Properties and Recyclability of Fiber Reinforced Polyester Composites
Source: ACS Sustain Chem Eng. 2024 Jun 20;12(26):10011–9. doi: 10.1021/acssuschemeng.4c03341 (PMC11220791; doi:10.1021/acssuschemeng.4c03341)
Supplement: Supplementary file 1 — sc4c03341_si_001.pdf [file sc4c03341_si_001.pdf]

Supporting Information for

Mechanical Properties and Recyclability of Fibre Reinforced Polyester Composites

Eloise K. Billington[a], Theona Şucu[a], Michael P. Shaver*[,a,b]

[a]Department of Materials, Engineering Building A, University of Manchester, Oxford Road, M13 9PL, U.K.;

[b]Sustainable Materials Innovation Hub, Henry Royce Institute, University of Manchester, Manchester, M13 9PL, U.K.

*michael.shaver@manchester.ac.uk

Pages - 12

Figures – 17

Schemes – 1

Tables – 3

Synthesis of salen ligand (N,N'-bis(salicylidene)-2,2-dimethyl-1,3-propanediamine)

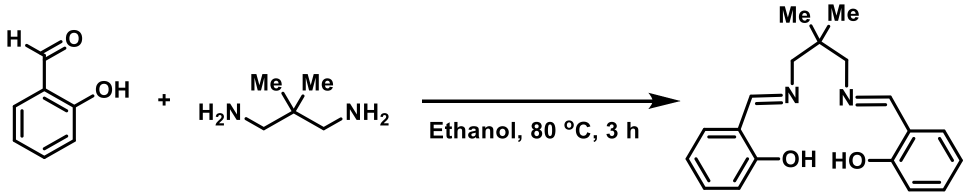

The salen ligand was prepared based on a previous literature procedure.[1] A solution of 2,2-dimethyl-1,3-propanediamine (10 g, 97.8 mmol) in ethanol was added to a vigorously stirring solution of salicylaldehyde (23.11 g, 195.7 mmol) in ethanol, at room temperature. The reaction mixture was heated to reflux (80 °C) and stirred for 3 h. The mixture was cooled on ice and the precipitated crystals were washed with cold ethanol, isolated by filtration and dried in vacuo, to afford yellow crystals (36.3 g, 79%). 1H NMR (CDCl3, 500 MHz): δ 8.34 (s, 2H), 7.36-7.24 (m, 2H), 6.99-6.96 (m, 2H), 6.92-6.86 (m, 2H), 3.49 (s, 4H), 1.08 (s, 6H).

Synthesis of salen aluminium pre-catalyst

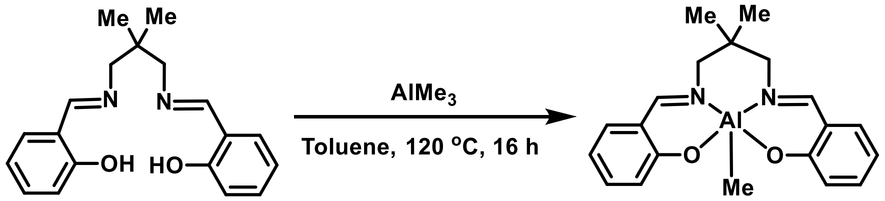

The catalyst was prepared based on a previous literature procedure.[1] In a nitrogen-filled glovebox, to a vigorously stirred solution of N,N'-bis(salicylidene)-2,2-dimethyl-1,3-propanediamine (4 g, 12.9 mmol) in toluene (60 mL) in a Schlenk flask, AlMe3 (6.45 mL of a 2.0 M solution in toluene, 12.9 mmol) was added, dropwise. The flask was sealed, removed from the glovebox and the reaction mixture was stirred at 110 °C for 16 h. Pale yellow crystals formed, which were washed with dry toluene and then dried under vacuum to afford the product (4.04 g, 89%). 1H NMR (CDCl3, 500 MHz): δ 8.10 (s, 2H), 7.40-7.33 (m, 2H), 7.23 (dd, 2H), 6.88-6.84 (m, 2H), 6.75-6.69 (m, 2H), 3.88 (d, 2H), 3.24 (d, 2H), 1.13 (s, 3H), 0.94-0.88 (s, 3H).

Synthesis of pre-initiated salen aluminium catalyst

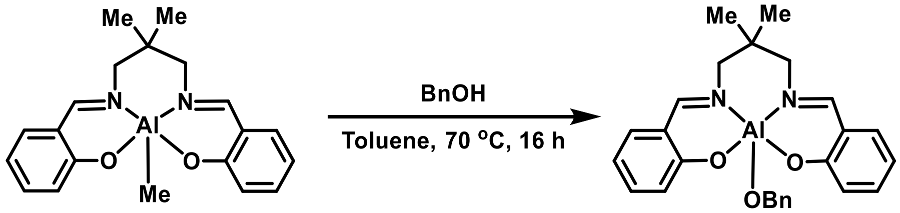

In a nitrogen-filled glovebox, to a vigorously stirred solution of pre-catalyst (3.48 g, 9.94 mmol) in toluene in a Schlenk flask, benzyl alcohol (1.18 g, 10.9 mmol) was added dropwise. The flask was sealed, removed from the glovebox and the reaction mixture was stirred at 70 °C for 16 h. White crystals were formed, which were washed with dry toluene and then dried under vacuum to afford the product (3.88 g, 88%). 1H NMR (CDCl3, 500 MHz): δ 8.10 (s, 2H), 7.40-7.33 (m, 2H), 7.23 (dd, 2H), 6.88-6.84 (m, 2H), 6.75-6.69 (m, 2H), 3.88 (d, 2H), 3.24 (d, 2H), 1.13 (s, 3H).

Synthesis of bisDOX ((4S,4'S)-[4,4'-bi(1,3-dioxolane)]-5,5'-dione) cross-linker

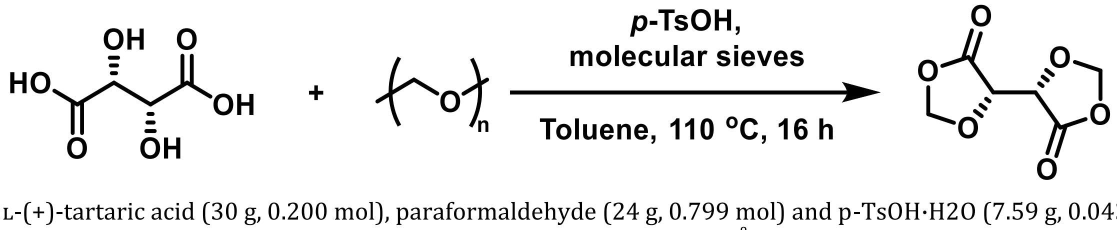

L-(+)-tartaric acid (30 g, 0.200 mol), paraformaldehyde (24 g, 0.799 mol) and p-TsOH·H2O (7.59 g, 0.043 mol) were dissolved in toluene (420 mL). Dried molecular sieves (4 Å) were added then the reaction mixture was heated to 110 °C and refluxed for 16 h. The mixture was cooled to room temperature, ethyl acetate (150 mL) was added, and the solution was washed successively with saturated $NaHCO_3$ solution

(300 mL), deionised water (300 mL) and saturated aqueous NaCl solution (300 mL). The organic layer was dried over $MgSO_4$, filtered under gravity and the solvent removed in vacuo to afford the crude product as a yellow oil. The residue was dissolved in THF, precipitated into hexane, filtered and dried in vacuo to afford white fluffy crystals (7.21 g, 21%). 1H NMR (CDCl3, 500 MHz): δ 5.61 (s, 2H), 5.57 (s, 2H), 4.71 (s, 2H).13C NMR (CDCl3, 126 MHz): δ 170.08, 95.97, 72.61.

Vacuum-Assisted Resin Infusion Methodology

The VARI set-up components include a layer of vacuum bagging, on top of which is laid the reinforcement, peel ply, perforated release film and infusion mesh, then finally a second layer of vacuum bagging, adhered to the first using sealant tape to maintain a closed system. Air is evacuated from the set-up using a vacuum pump, then the liquid resin is drawn through under vacuum and infuses into the preform until complete impregnation is achieved. Finally, the composite is cured and removed from the set-up.

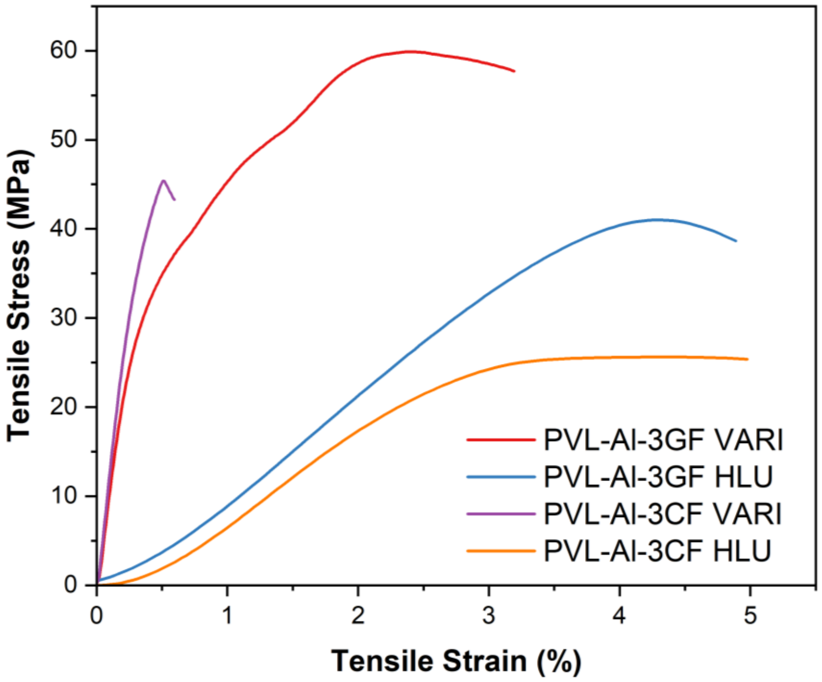

Figure S1: Tensile plot of **PVL-Al-3GF** composites synthesised using hand lay-up (HLU) vs vacuum-assisted resin infusion (VARI) methodology.

Table S1: Mechanical properties of PVL-Al composites with glass fibre reinforcements, manufactured using hand lay-up. [a]σ_b (stress at break), ε_b (strain at break) and E (Young's modulus) data obtained from tensile testing measurements, [b]Fibre content obtained from TGA analyses.

Sample	σ_b (MPa)[a]	ε_b (%)[a]	E (GPa)[a]	Fibre content (%)[b]
**PVL-Al-3GF**	**43.9 ± 8.1**	**5.82 ± 1.22**	**1.12± 0.18**	**30.8 ± 2.1**
**PVL-Al-3NH_2mGF**	**49.1 ± 15.5**	**7.20 ± 2.81**	**1.13 ± 0.25**	**36.9 ± 3.9**
**PVL-Al-3EtmGF**	**74.8 ± 3.3**	**18.8 ± 3.18**	**1.03 ± 0.10**	**47.0 ± 2.7**
**PVL-Al-3epoxymGF**	**39.6 ± 12.9**	**7.75 ± 1.86**	**0.97 ± 0.25**	**32.5 ± 3.8**
**PVL-Al-3(10:90 NH_2/Et)mGF**	**24.1 ± 11.1**	**5.23 ± 0.64**	**0.67 ± 0.19**	**38.7 ± 1.3**
**PVL-Al-J-EtmGF**	**76.1 ± 14.4**	**8.69 ± 1.26**	**1.43 ± 0.27**	**33.8 ± 1.5**

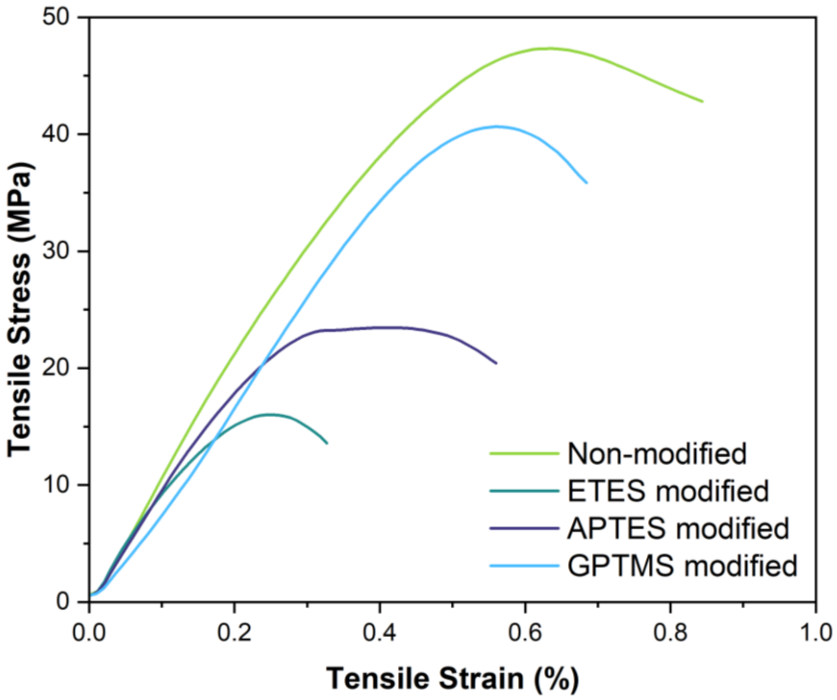

Figure S2: Tensile plot of **PVL-Al-1GF** composites with modified glass fibre reinforcement, synthesised using vacuum-assisted resin infusion (VARI) methodology.

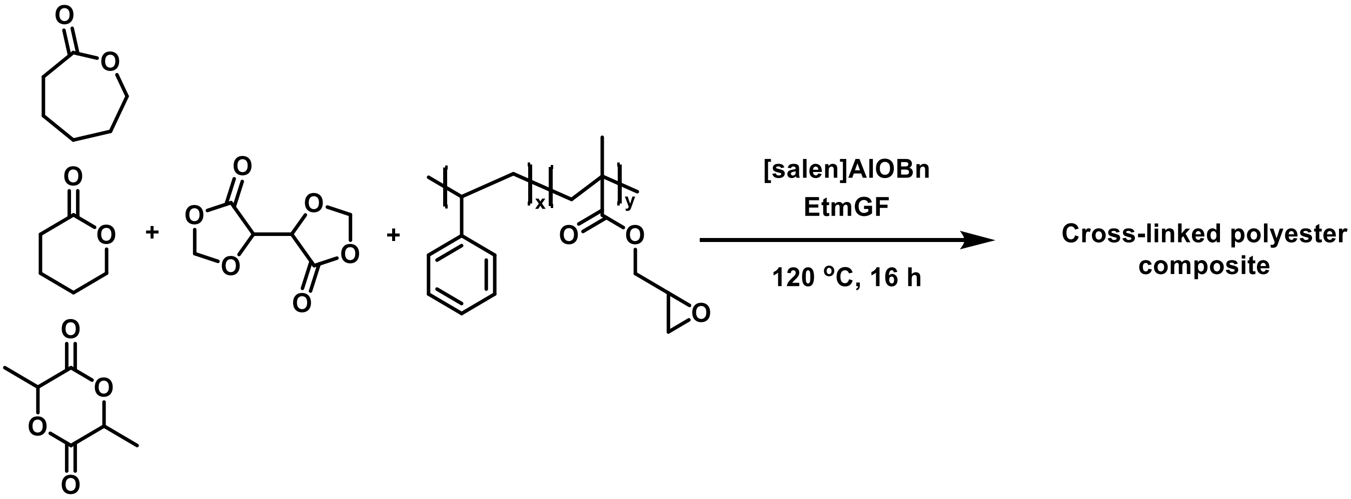

Scheme S1: Synthesis of polyester composites with Joncryl ADR-4400 additive.

Table S2. Thermal properties of PVL-Al, PCL-Al, PLA-Al, PVL-Sn and PLA-Sn composites with glass and carbon fibre reinforcements. [a]Glass transition temperature (T_g), melting temperature (T_m) and enthalpy of melting (ΔH_m) obtained from the second heating ramp of a DSC experiment. [b]Crystallisation temperature (T_c) and enthalpy of crystallisation (ΔH_c) obtained from the first cooling ramp of a DSC experiment. [c]Degradation temperature 5% mass loss ($T_{d,5\%}$) obtained from TGA analyses.

Sample	T_g (°C)[a]	T_m (°C)[a]	ΔH_m (J/g)[a]	T_c (°C)[b]	ΔH_c (J/g)[b]	$T_{d,5\%}$ (°C)[c]
**PVL-Al-3GF**	**-**	**28.9 ± 2.8**	**7.2 ± 1.1**	**-8.20 ± 0.42**	**-3.71 ± 0.42**	**248 ± 4**
**PCL-Al-3GF**	**-**	**31.1 ± 0.7**	**9.03 ± 0.03**	**11.1 ± 0.8**	**7.72 ± 0.26**	**253 ± 9**
**PLA-Al-3GF**	**25.1 ± 5.2**	**-**	**-**	**-**	**-**	**247 ± 0**
**PVL-Sn-3GF**	**-**	**29.8 ± 0.2**	**13.0 ± 0.8**	**2.02 ± 0.44**	**11.9 ± 0.8**	**235 ± 2**
**PLA-Sn-3GF**	**44.2 ± 1.6**	**-**	**-**	**-**	**-**	**233 ± 6**
**PVL-Al-3CF**	**-43.8 ± 0.7**	**33.5 ± 0.4**	**12.5 ± 0.2**	**-2.70 ± 1.14**	**10.8 ± 0.6**	**248 ± 1**
**PLA-Al-3CF**	**34.2 ± 5.4**	**-**	**-**	**-**	**-**	**219 ± 6**
**PVL-Sn-3CF**	**-**	**26.5 ± 0.3**	**19.2 ± 0.2**	**-4.99 ± 0.51**	**18.1 ± 0.5**	**208 ± 1**
**PLA-Sn-3CF**	**26.7 ± 7.9**	**-**	**-**	**-**	**-**	**224 ± 5**

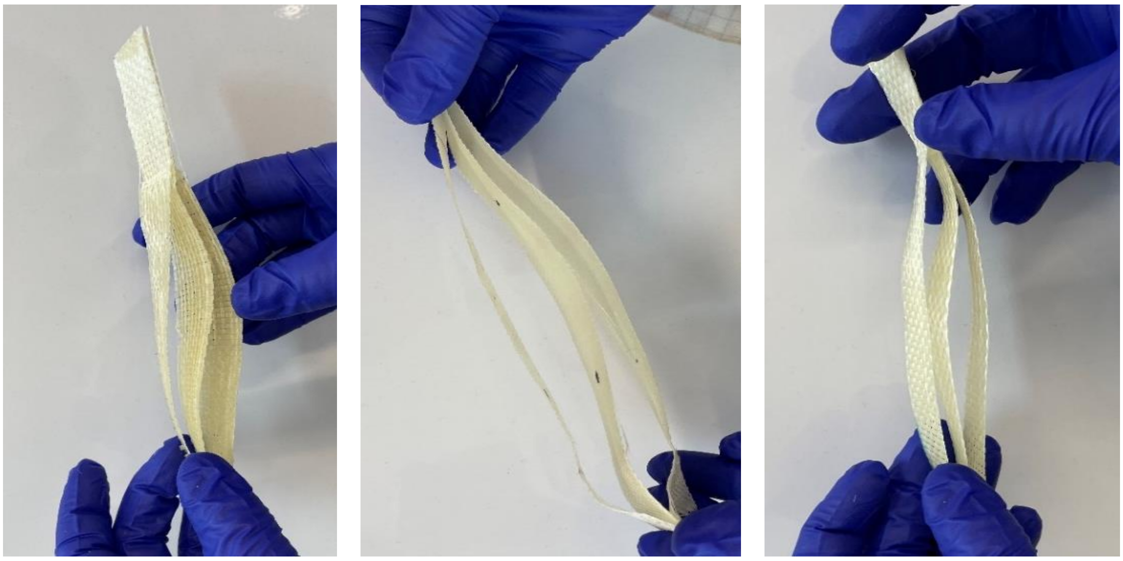

Figure S3: Picture of delaminated **PVL-1-3GF** tensile testing sample.

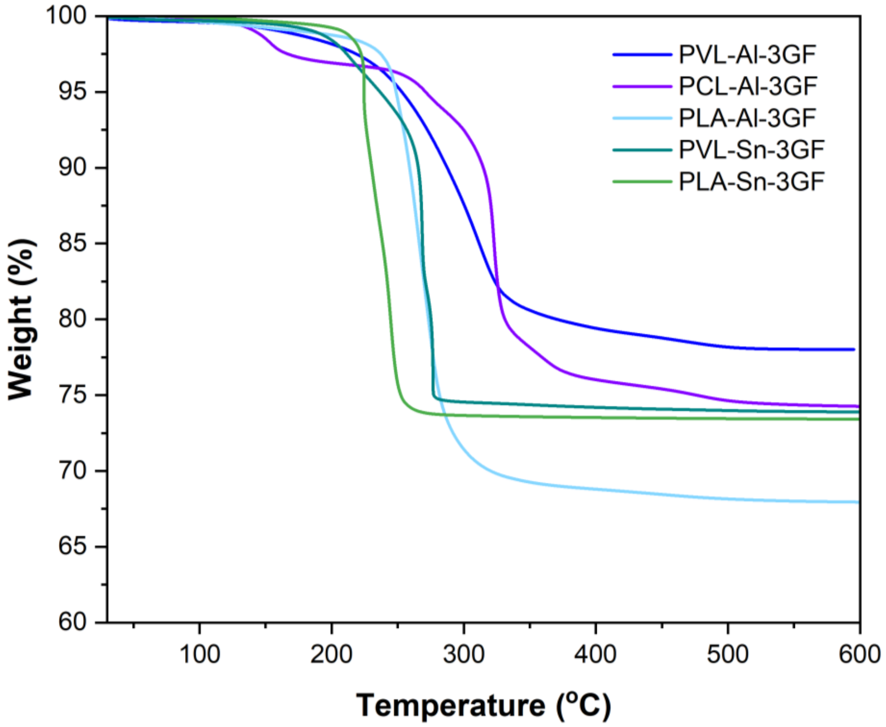

Figure S4: Thermogravimetric analysis (TGA) of **PCL**, **PVL** and **PLA** GF composites.

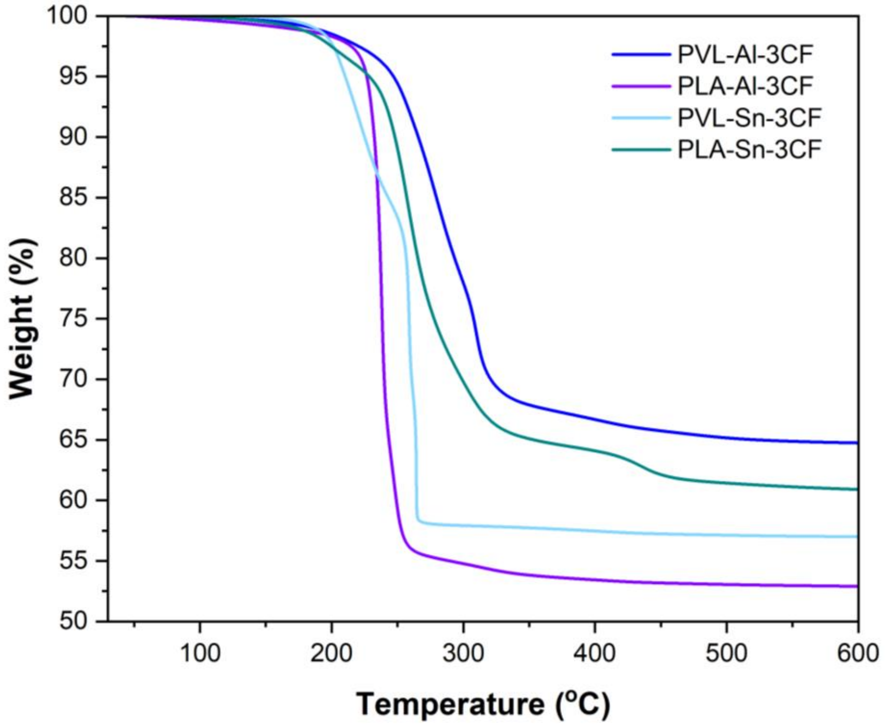

Figure S5: Thermogravimetric analysis (TGA) of PCL, PVL and PLA CF composites.

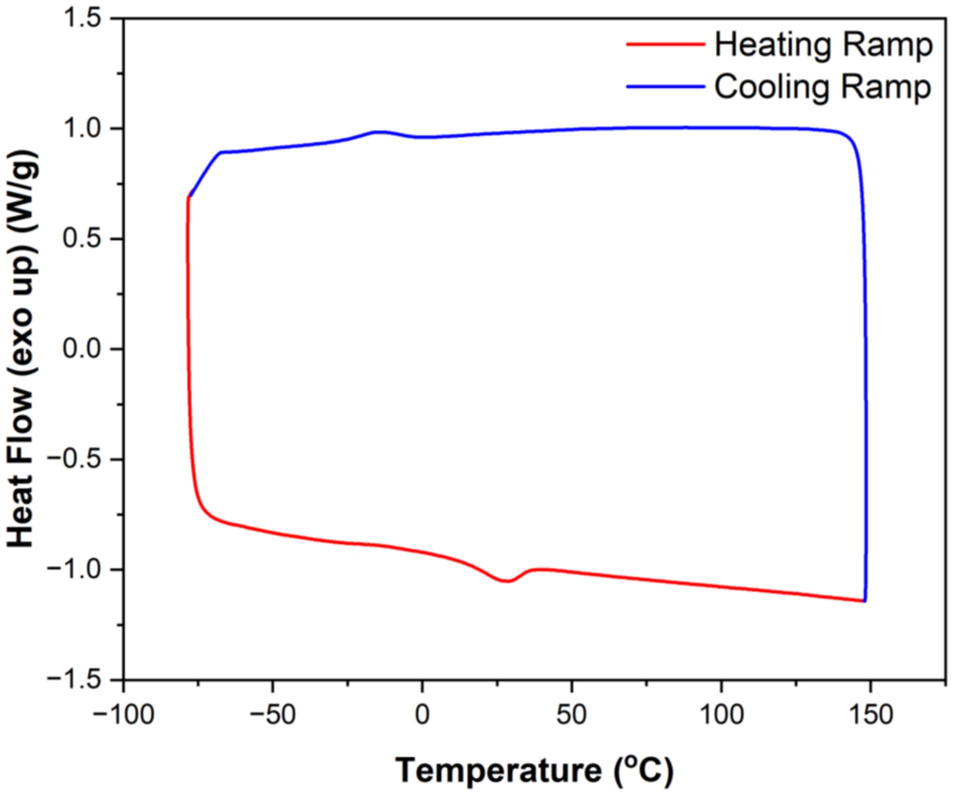

Figure S6: Differential scanning calorimetry (DSC) trace for **PVL-Al-3GF,** with second heating and cooling.

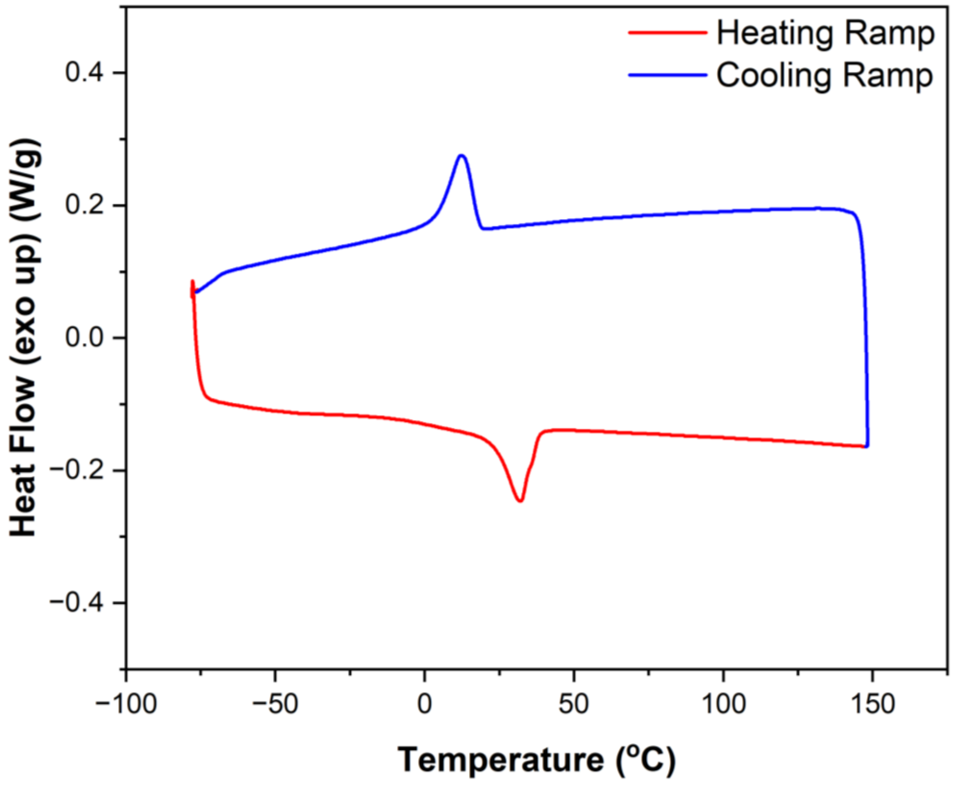

Figure S7: Differential scanning calorimetry (DSC) trace for **PCL-Al-3GF**, with second heating and cooling.

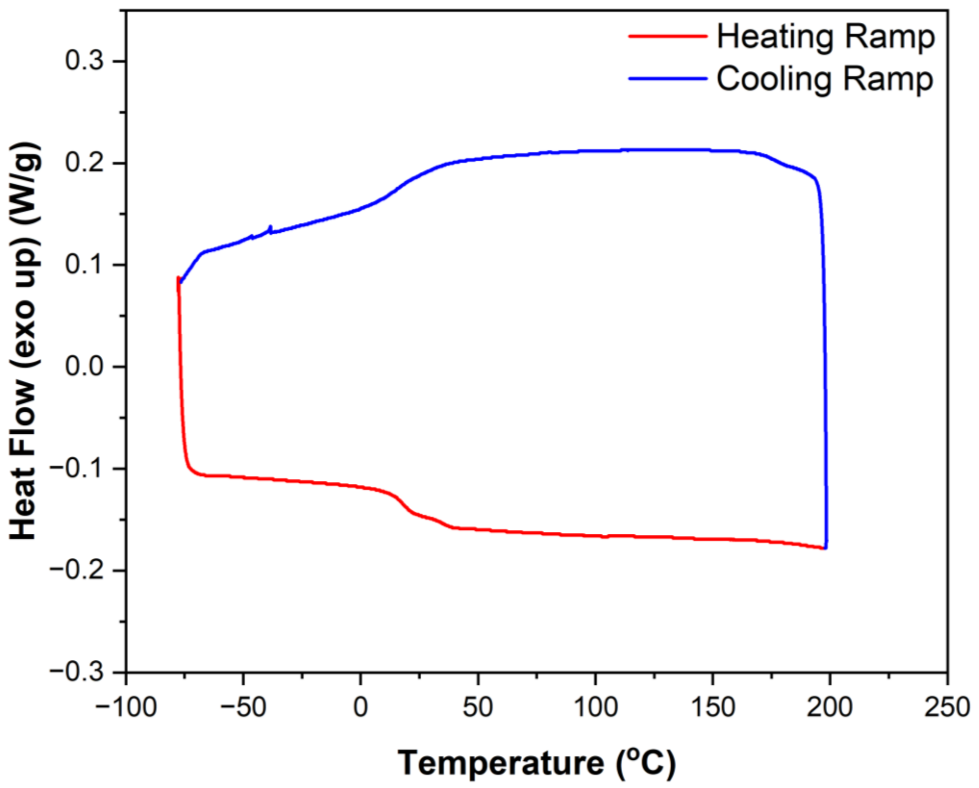

Figure S8: Differential scanning calorimetry (DSC) trace for **PLA-Al-3GF**, with second heating and cooling.

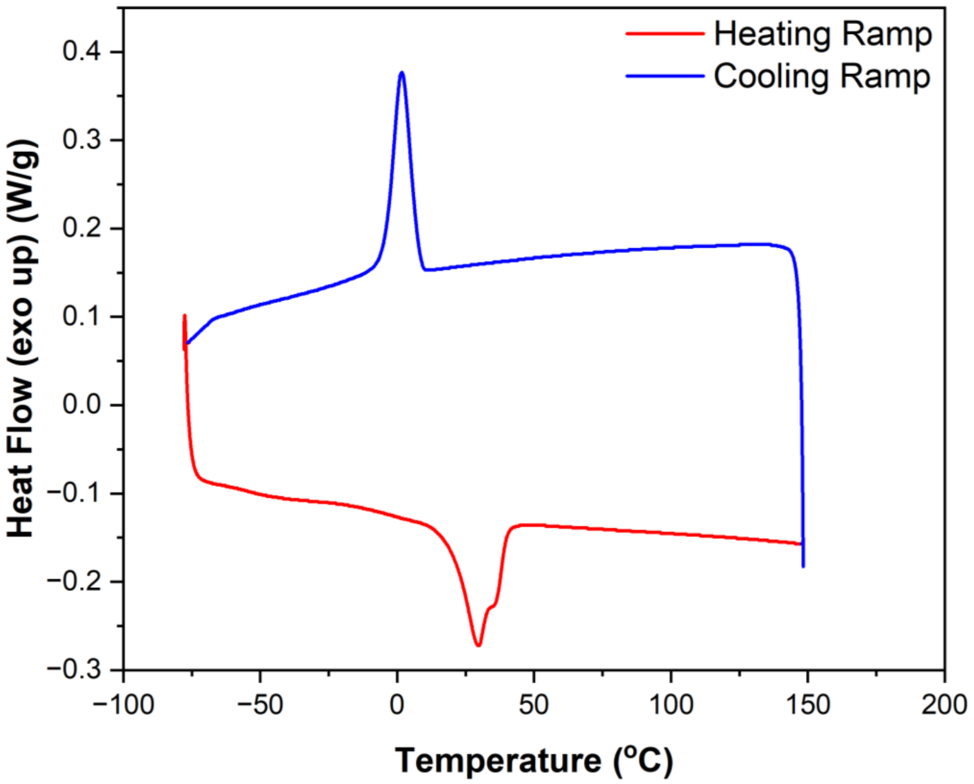

Figure S9: Differential scanning calorimetry (DSC) trace for **PVL-Sn-3GF**, with second heating and cooling.

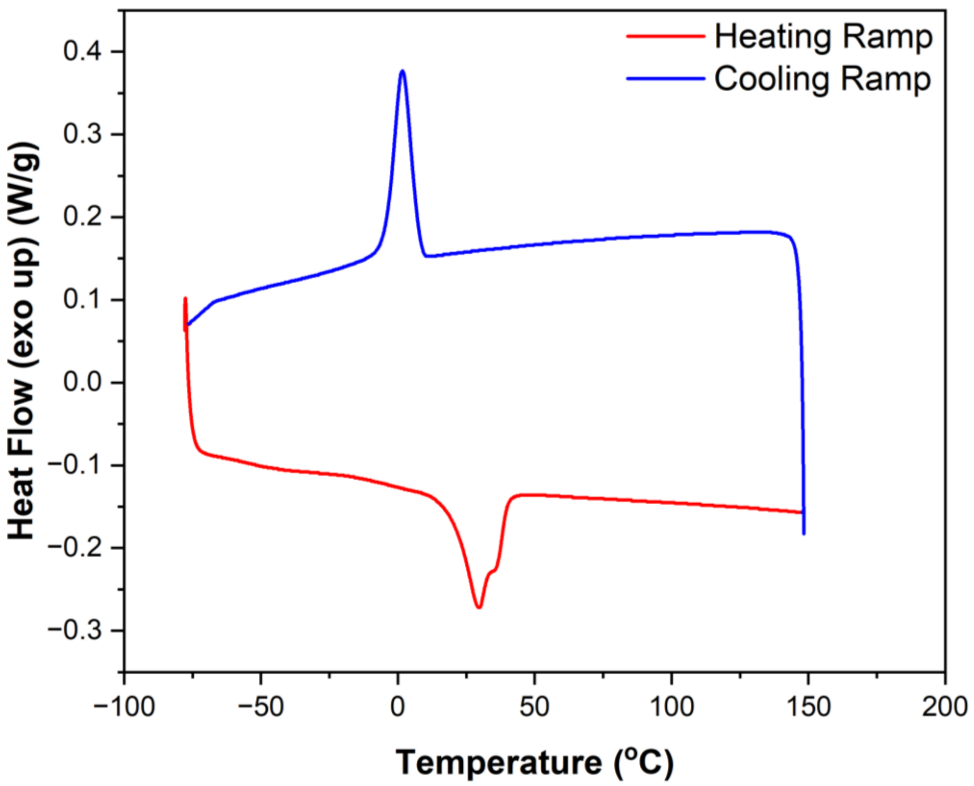

Figure S10: Differential scanning calorimetry (DSC) trace for **PLA-Sn-3GF**, with second heating and cooling.

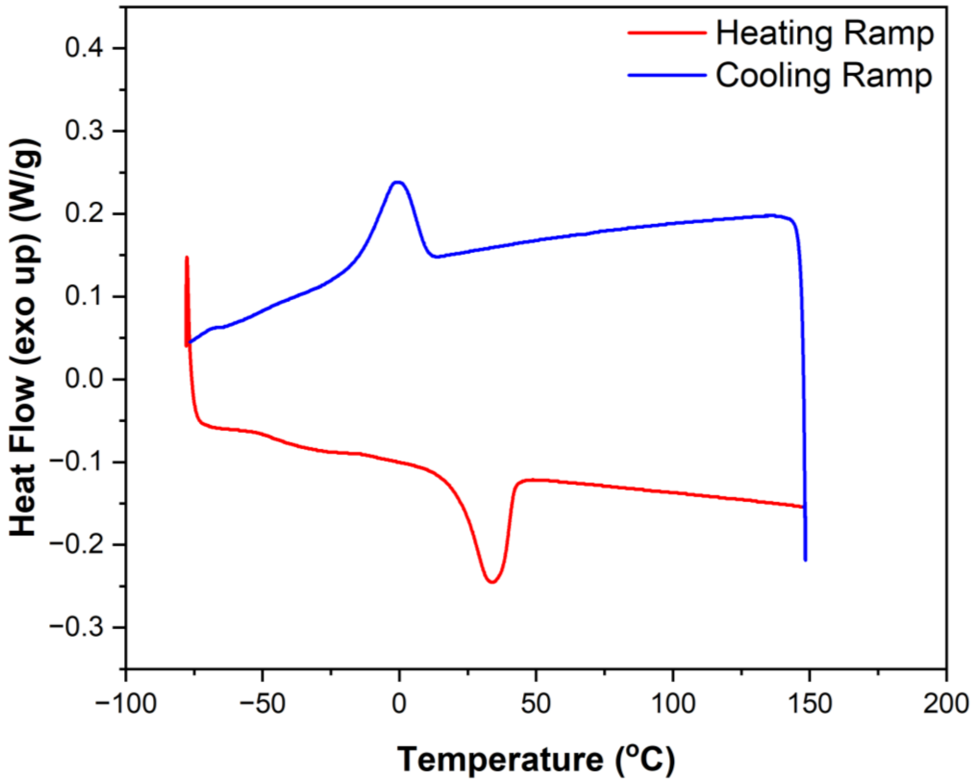

Figure S11: Differential scanning calorimetry (DSC) trace for **PVL-Al-3CF**, with second heating and cooling.

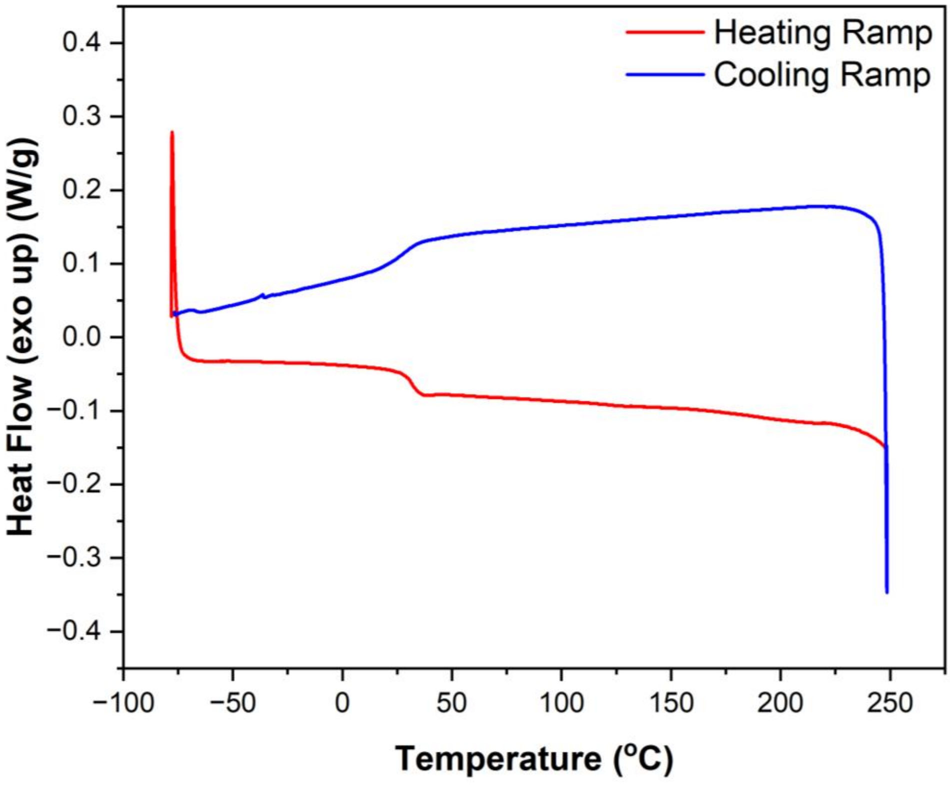

Figure S12: Differential scanning calorimetry (DSC) trace for **PLA-Al-3CF**, with second heating and cooling.

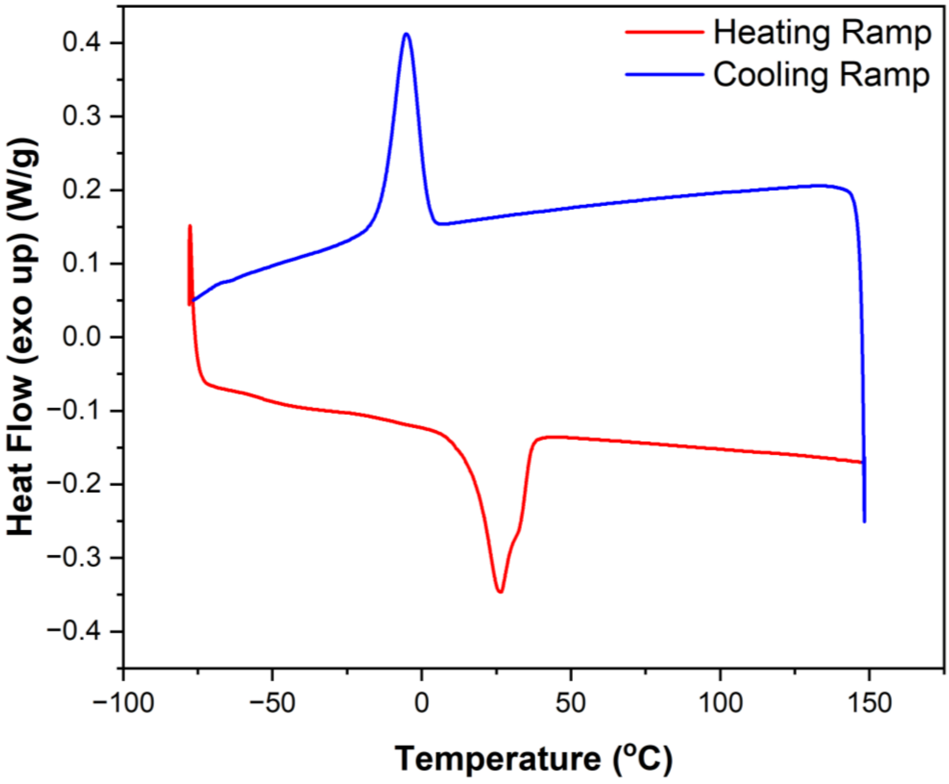

Figure S13: Differential scanning calorimetry (DSC) trace for **PVL-Sn-3CF**, with second heating and cooling.

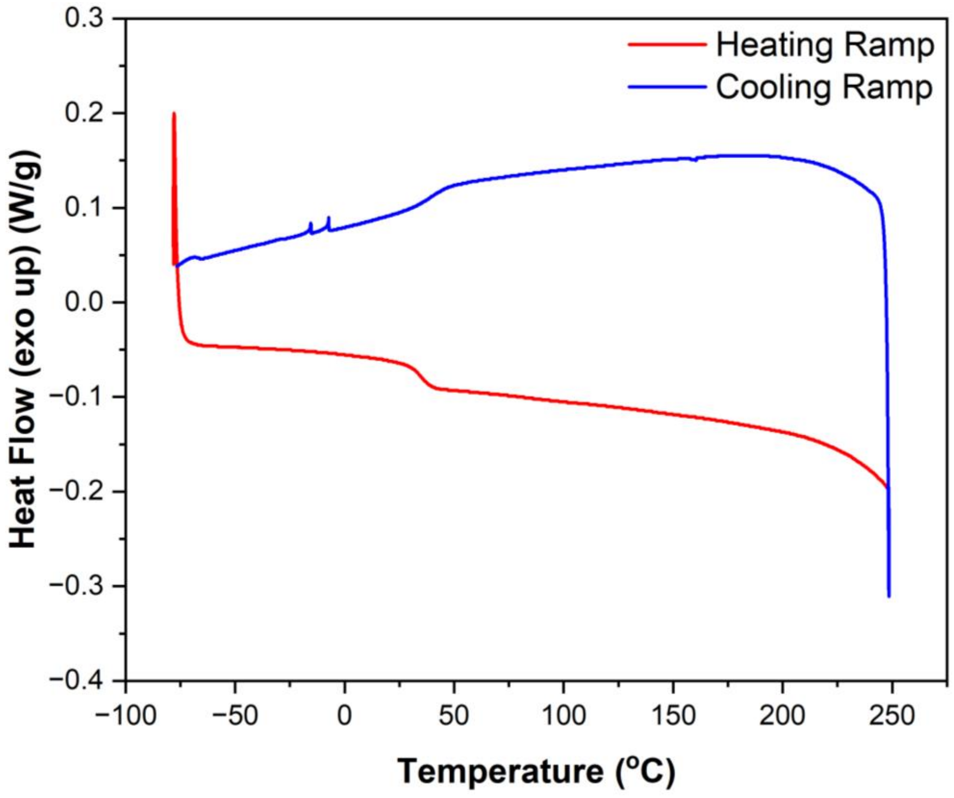

Figure S14: Differential scanning calorimetry (DSC) trace for **PLA-Sn-3CF**, with second heating and cooling.

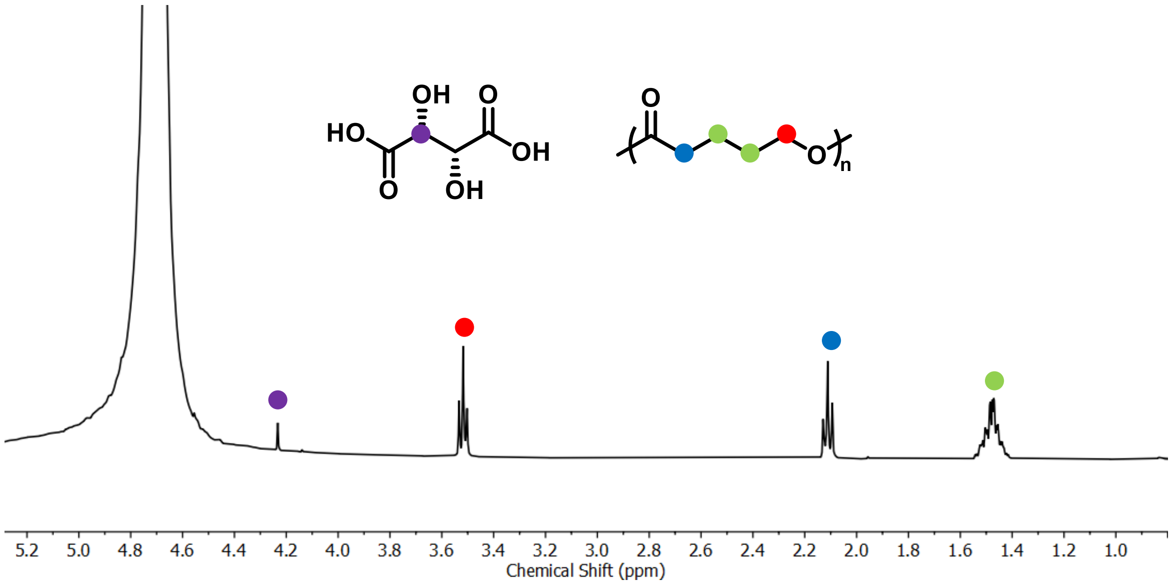

Figure S15: ^{1}H NMR spectrum (D_2O, 400 MHz) of the **PVL-Al-3GF** degradation solution.

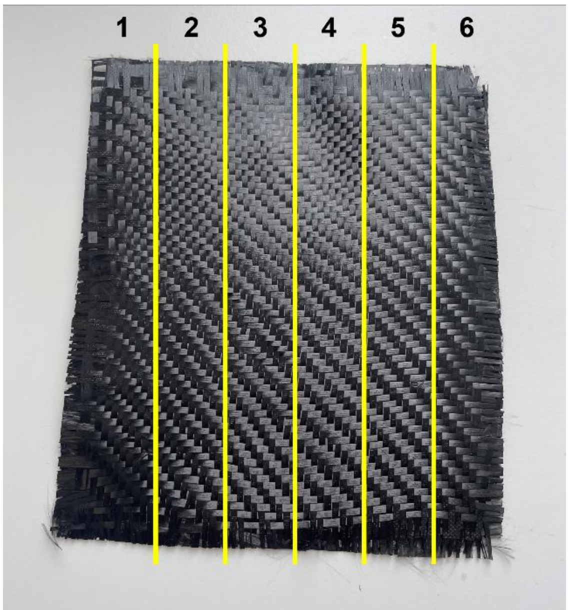

Figure S16: Picture of **PLA-Al-3rCF** indicating middle (2-5) and edge (1 & 6) tensile testing samples.

Table S3: Mechanical properties of PLA-Al composites with carbon fibre reinforcements, pre and post recycling, manufactured using compression moulding. σ_b (stress at break), ε_b (strain at break) and E (Young's modulus) data obtained from tensile testing measurements.

Sample	σ_b (MPa)	ε_b (%)	E (GPa)
**PLA-Al-3CF**	202 ± 15	1.75 ± 0.21	24.5 ± 1.7
**PLA-Al-3CF middle**	154 ± 14	1.76 ± 0.11	16.5 ± 1.7
**PLA-Al-3CF edge**	55 ± 11	4.95 ± 0.33	7.7 ± 2.8

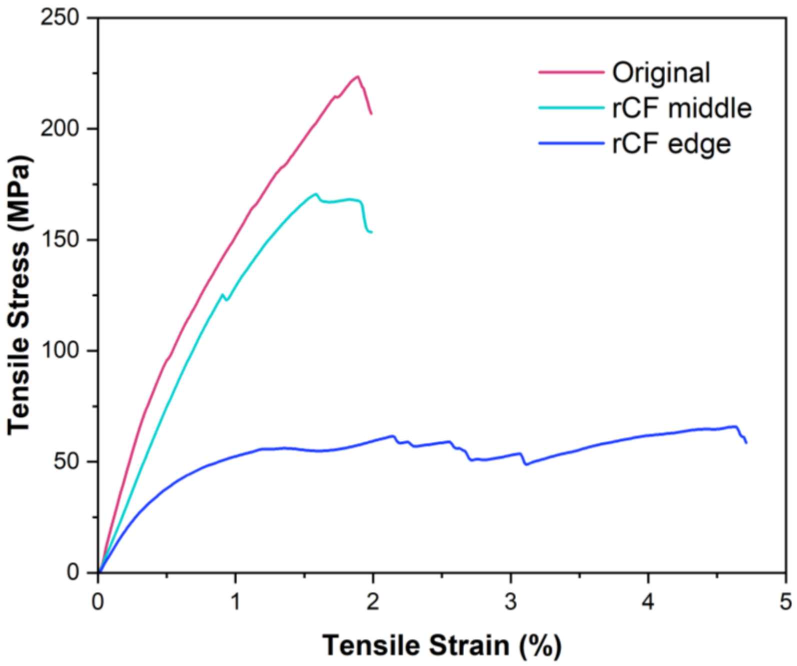

Figure S17: Tensile plot for original (**PLA-Al-3CF**) and recycled (**PLA-Al-3rCF**) PLA composites, manufactured *via* compression moulding.

References:

(1) Hormnirun, P.; Marshall, E. L.; Gibson, V. C.; Pugh, R. I.; White, A. J. P. Study of Ligand Substituent Effects on the Rate and Stereoselectivity of Lactide Polymerization Using Aluminum Salen-Type Initiators. *Proc. Natl. Acad. Sci. U. S. A.* **2006**, *103* (42), 15343–15348. https://doi.org/10.1073/pnas.0602765103.
